# *Platycodon grandiflorum* exhibits anti-neuroinflammatory potential against beta-amyloid-induced toxicity in microglia cells

**DOI:** 10.3389/fnut.2024.1427121

**Published:** 2024-08-07

**Authors:** Yun-Jeong Ji, Min Hye Kang, Geum-Soog Kim, Hyung Don Kim, Gwi Yeong Jang

**Affiliations:** ^1^Department of Herbal Crop Research, National Institute of Horticultural Herbal Science, Rural Development Administration, Eumseong, Republic of Korea; ^2^Department of Biochemistry, School of Life Sciences, Chungbuk National University, Cheongju, Republic of Korea

**Keywords:** *Platycodon grandiflorum*, amyloid beta, microglia cells, neuro-inflammatory, NF-κB pathway

## Abstract

**Background/objectives:**

*Platycodon grandiflorum* (PG) is used in traditional oriental medicine to treat several ailments.

**Methods:**

The study investigated the anti-inflammatory and neuroprotective effects of PGW (*P. grandiflorum*) extract in Aβ25-35-induced inflammation in BV2 microglia cells.

**Result:**

PGW demonstrated significant inhibition of nitric oxide (NO) production, with reductions of 30.4, 36.7, and 61.2% at concentrations of 50, 100, and 200 μg/mL, respectively. Moreover, PGW effectively suppressed the production of pro-inflammatory cytokines IL-1β and IL-6 and exhibited significant inhibitory activity against TNF-α at 200 μg/mL. Furthermore, PGW treatment mitigated apoptosis in Aβ-induced BV2 cells by modulating the mitochondrial apoptosis pathway, regulating Bcl-2 family protein synthesis, and inhibiting caspase activation. Mechanistically, PGW attenuated the activation of the MAPK (JNK, ERK, p38) pathway induced by Aβ, showing a concentration-dependent decrease in phosphorylation levels of these proteins. Additionally, PGW inhibited the NF-κB pathway activation by reducing the phosphorylation levels of p65 and IκBα in a concentration-dependent manner.

**Conclusion:**

PGW demonstrated anti-inflammatory and neuroprotective effects in Aβ-induced neuronal cells, suggesting its potential as a therapeutic agent for neuroinflammatory associated with neurodegenerative diseases.

## 1 Introduction

*Platycodon grandiflorum* (PG) is a medicinal plant with a long history in Korea, China, and Japan. It has been used for various traditional medical purposes (1). PG root is particularly effective in treating bronchial diseases, coughs, colds, and indigestion. These traditional effects are attributed to the plant’s triterpenoid saponins, carbohydrates, and fiber, especially saponins and platycodins (2). PG can lower blood sugar (3), improves cholesterol metabolism (4), exerts anti-obesity (5), anti-inflammatory (6, 7), immunostimulatory (2), and antioxidant effects (8), and ameliorates atopic dermatitis (9). Based on traditional efficacy and modern scientific research results, bellflower root extract’s beneficial effects have been proven in the peripheral nervous system and the brain. PG extract showed a protective effect against Aβ25-35-induced neurotoxicity, which prevents neuronal cell death by inhibiting the production of reactive oxygen species. Based on this accumulated evidence, many studies have recently reported the efficacy of bellflower root extract in Alzheimer’s disease. Our previous study reported that PGE reduces oxidative stress and inhibits Aβ deposition in the hippocampus of AD animal models. Additionally, saponin, an essential component of PGE, not only alleviated oxidative stress and prevented neuronal cell death in Aβ-treated hippocampal HT22 cells but also prevented AD-related pathologies such as Aβ deposition, oxidative damage, neuroinflammation, and neurodegeneration in AD animal models (10). Although PG has shown beneficial effects in AD, the mechanisms underlying PGE’s effects in the brain remain unclear, particularly with regard to its effects on oxidative stress, neuroinflammation, and Aβ deposition.

Many studies have been conducted to elucidate the relationship between Aβ and neuroinflammation in the pathogenesis of Alzheimer’s disease (11). Because inflammatory mechanisms are highly interactive and rarely occur in isolation, anti-inflammatory effects may alleviate various diseases or symptoms resulting from Alzheimer’s disease (12). Although the mechanisms by which Aβ triggers inflammatory processes are complex, there is evidence that this peptide induces activation of the transcription factor NF-κB. In response to inflammatory stimuli, IκB kinase (IKK) phosphorylates IκB inhibitors and free NF-κB translocates to the nucleus and binds to κB binding sites in the promoter regions of target genes. This induces the transcription of proinflammatory mediators such as inducible nitric oxide synthase (iNOS), cyclooxygenase-2 (COX-2), tumor necrosis factor-α (TNF-α), and interleukin-1β (IL-1β) (13). Also, mitogen-activated protein kinases (MAPKs) regulate the production of key inflammatory mediators. Upon exposure to Aβ stimulation, c-Jun N-terminal kinase (JNK), extracellular signalregulated kinase (ERK), and p38 MAPK are activated by phosphorylation, which regulates the activation of the NF-κB signaling pathway (14). These findings suggest that Aβ-induced neuroinflammation is associated with activating NF-κB and MAPK signaling pathways, leading to the expression of inflammatory mediators and potentially contributing to AD pathogenesis (15, 16). As a result, increased Aβ accumulation stimulates the aging of neurons and microglia, which promotes neuroinflammation and neurodegeneration, resulting in a vicious cycle of Alzheimer’s disease.

Most studies mainly focus on the neuroprotective effects of PG extracts on neurons, and little is known about the mechanisms by which PG extracts protect glial cells from Aβ-induced neurotoxicity. In this study, to investigate the anti-inflammatory and neuroprotective effects of PGW on BV2 microglial cells, oxidative stress, and inflammation were induced by treating BV2 microglial cells with Aβ25-35. We investigated whether PGW could inhibit ROS and nitric oxide (NO) production in BV2 microglia under oxidative stress. Specifically, we evaluated PGW’s nitric oxide (NO) inhibitory effect in BV2 microglial cells treated with Aβ25-35. Additionally, we determined the impact of PGW on Aβ-induced ROS generation and toxicity in Aβ25-35-treated BV2 microglia. Next, we investigated the effect of PGE, which showed a nitric oxide (NO) inhibitory effect, on proinflammatory cytokine production in Aβ-treated BV2 microglial cells. Finally, we sought to demonstrate how PGW alleviates neuroinflammation and exerts neuroprotective effects by activating NF-κB and MAPK signaling pathways in Aβ-induced neurons.

## 2 Materials and methods

### 2.1 Sample preparation and extraction

The‘Etteumbeak’ PG used in this study was harvested in Boeun, Chungcheongbuk-do in 2022. ‘Etteumbeak’ PG has been altered from diploid to tetraploid by treating native PG with colchicine. ‘Etteumbeak’ PG grows rapidly and can be cultivated for 1–2 years, and its productivity is ~40% higher than native PG. Fifty liters of water (sample: solvent, 1:10) per 5 kg of PG sample were added, and hot-water extraction was performed at 90°C for 6 h. The extract was filtered and lyophilized to prepare a dry base. The extraction yield was 34%, the sample (PGW) was dissolved in dimethylsulfoxide (DMSO; Sigma-Aldrich, St. Louis, MO, United States) and stored at −80°C until needed.

### 2.2 Analysis of saponins from PGW by HPLC-UVD

After dissolving PG extract in 40 mL of distilled water, it was degreased with diethyl ether using a separatory funnel. Afterward, the separated aqueous layer was extracted three times using n-butanol saturated with water. The n-butanol layer was evaporated at 50°C to obtain a residue, which was dissolved in methanol and used for analysis. Saponin standards (Platycoside G1, Platycoside E, Deapio-platycodin D3, Platycodin D3, Deapio-platycodin D, Platyconic acid A, Platycodin D2, Platycodin D, Polygalacin D, Platycoside A, Platycodin A, Platycodigenin) were obtained from Chengdu Biopurify Phytochemicals Ltd. (Chengdu, Sichuan, China). HPLC-grade water and acetonitrile (ACN) were purchased from J.T. Baker (Phillipsburg, NJ, United States). All other chemicals were of reagent grade. Methanol (1 mL) was added to the PGW powder 100 mg, and the mixture was vortexed and filtered through a membrane filter (0.45 μm) for using an analytical sample. Saponins were analyzed by high-performance liquid chromatography (HPLC; 1,200 Series, Agilent Technologies, Santa Clara, CA, United States). Under separation conditions, the mobile phase consisted of 0.1% formic acid in water (solvent A) and 0.1% formic acid in ACN (solvent B), with gradient elution: 0–5 min, isocratic 10% A; 5–19 min, 10–22% B; 19–37 min, 22–28% B; 37–54 min, 28–35% B; 54–59 min, 35–60% B; 59–60 min, and 60–95% B. A Triart C18 column (100 × 4.6 mm, 3 μm i.d.; YMC Co., Kyoto, Japan) was used, with a temperature of 30°C and flow rate of 1 mL/min. The diode-array detection detector wavelength was 203 nm. The sample injection volume was 30 μL, and the column temperature was maintained at 40°C. The ELSD conditions were set to an atomizer temperature of 42°C, drift tube temperature of 85°C, and N2 gas pressure of 50 psi for the analysis.

### 2.3 Cell culture

Mouse brain-derived BV2 microglial cells were cultured in Dulbecco’s modified Eagle’s medium (DMEM; Gibco, Canada) containing 1% penicillin/streptomycin and 10% fetal bovine serum (FBS) at 37°C in an atmosphere containing 5% CO2. Subcultures were performed every 2 days and cells at passages 5–10 were used in experiments.

### 2.4 Cell viability assay

BV-2 cells are a cell line that replaces microglia. They will reflect the characteristics of microglia and are suitable for evaluating the role of microglia in eliminating Aβ toxic accumulation in the early stages of Alzheimer’s disease (17). In support, we assessed neuroinflammation in response to Aβ-induced toxicity in BV-2 cells (microglia). To investigate the effect of PGW on Aβ25-35-induced cytotoxicity, BV2 microglial cells were treated with Aβ25-35, alone (10 mM; Sigma-Aldrich) or with PGW for 24 h (alone) or 1 h (with PGW), followed by Aβ for 24 h. Next, the cells were treated with 3-(4,5-dimethylthiazol-2-yl)-5-(3-carboxymethoxyphenyl)-2-(4-sulfophenyl)-2H-tetrazolium(MTS; Promega, Madison, WI, United States) and the change in absorbance at 490 nm was measured using a multiplate reader (Biotek).

### 2.5 Measurement of NO production

To assay NO production, BV2 microglia were transferred to 96-well plates at 1.0 × 10^4^/well and, 24 h later, were pretreated with PGW extract (0–200 μg/mL) for 1 h. Following 18 h of treatment with 10 μM/mL Aβ, equal volumes of culture medium and Griess reagent (Promega) were added to the 96-well plate, followed by for 10 min. Absorbance at 540 nm was measured using a multiplate reader.

### 2.6 Enzyme-linked immunosorbent assay

BV2 microglial cells (1.0 × 10^5^ cells/mL) were distributed on 60 mm plates, cultured for 24 h, and then pretreated with PGW (50,100 and 200 μg/mL). After 1 h, cells were treated with ab (10 μg/mL) and cultured for 24 h. Afterward, the culture medium was centrifuged at 10,000 rpm for 3 min to remove precipitates, and the supernatant was recovered. The levels of TNF-α, IL-6, and IL-1β in the recovered supernatant were measured using a mouse enzyme-linked immunosorbent assay (ELISA) kit (R&D Systems Inc., Minneapolis, MN, United States). Next, the membrane was blocked with 2% BCS for 1 h and washed three times with TBST solution, once every 10 min.

### 2.7 Preparation of cell lysates and western blotting

BV2 cells were seeded on a plate at 1.0 × 104/well, incubated for 24 h, treated with PGW at 50, 100, and 200 μg/mL for 1 h, and treated with Aβ25-35 at 10 μM for 48 h. Next, sample was washed twice with phosphate-buffered saline (PBS), dissolved in radioimmunoprecipitation assay (RIPA) buffer (Cell Signaling Technology, Danvers, MA, United States), and reacted on ice for 30 min. The cell lysis solvent was centrifuged at 4°C and 13,000 rpm for 20 min, and the protein concentration in the supernatant was quantified using Bradford’s reagent (Bio-Rad, Hercules, CA, United States). The cell lysis solvent was mixed with 4× Laemmli buffer (iNtRON, Seongnam, South Korea) and heated at 95°C for 5 min, and samples corresponding to 10 μg of protein were separated by 10% SDS-PAGE. The proteins were transferred to a polyvinylidene difluoride membrane (PVDF; Millipore, Darmstadt, Germany) and blocked with 2% bovine serum albumin in TBST at room temperature for 30 min. Primary antibodies against tyrosinase (TRP-1 and TRP-2; Cell Signaling Technology) were diluted 1:1,000, added to the membrane, and incubated overnight at 4%. Subsequently, the membrane was washed thrice with TBST for 10 min each, and the secondary antibody (Cell Signaling Technology; 1:2,000), was added over 1 h. The membrane was washed three times with TBST for 10 min each. Proteins were detected using the Enhanced Chemiluminescence Western Blotting Detection Kit (Bio-Rad) and the ChemiDoc Imaging System (Bio-Rad).

### 2.8 Statistical analysis

All analyses were randomly conducted in a blinded manner. Statistical analyses were conducted using GraphPad Prism 7.0 software (GraphPad Software, La Jolla, CA, United States). Data are presented with error bars representing standard deviations (SD). Nonlinear regression analysis was performed to derive curves showing the relationship between each group. The significance of differences between groups was evaluated using an independent t-test and one-way ANOVA, followed by Tukey’s *post-hoc* test or Fisher’s LSD test for pairwise comparisons. Effect sizes were included to measure the magnitude of observed differences to provide a more comprehensive statistical analysis. For t-tests, Cohen’s d was calculated, and for ANOVA, eta-squared (η^2^) values were reported. Additionally, 95% confidence intervals (CIs) were provided for mean differences and effect sizes to assess the recision and reliability of the estimates. Statistical significance was considered at *p* < 0.05.

## 3 Results

### 3.1 HPLC analysis of *Platycodon grandiflorum* water extract

**Figure 1:**
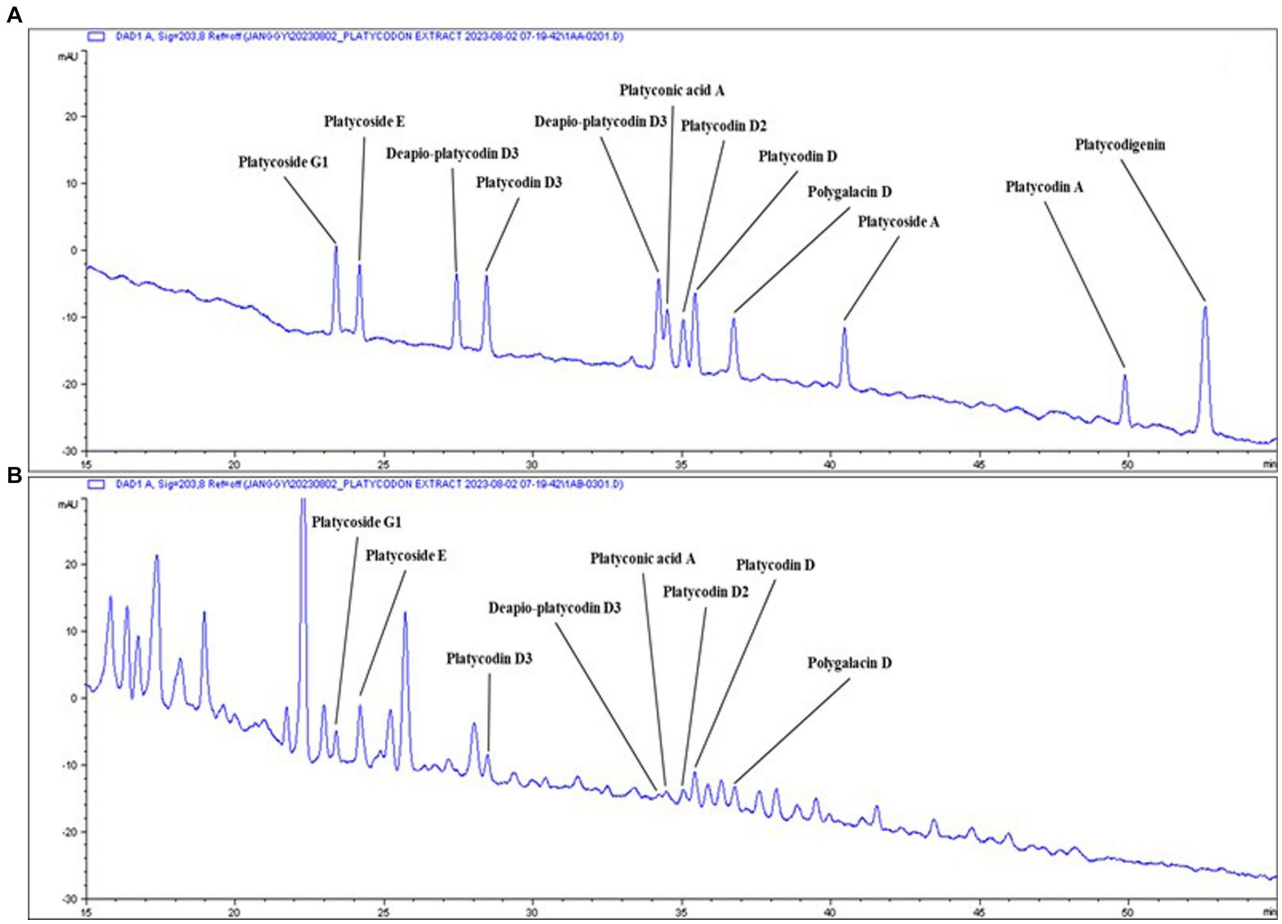
Typical chromatogram of PGW. Samples: **(A)** Saponin standards, **(B)** *Platycodon grandiflorum* water extract (PGW).

Chromatograms of PG saponin standards and PGW are depicted in Table 1 and Figure 1. By comparing retention times (RT) with 12 saponin standards, including major saponins such as platycoside E and platycodin D (commonly known as PG saponins), eight saponins were identified in PGW. The elution RT confirmed the presence of platycoside G1 (deapi-platycoside E), platycoside E, platycodin D3, deapio-platycodin D3, platyconic acid A, platycodin D2, platycodin D2, platycodin D, and polygalacin D. The contents of these saponins were measured at 292.56, 801.72, 270.99, 84.45, 194.65, 326.03, 381.77, and 337.76 μg/g, respectively, with Platycoside E exhibiting the highest concentration.

**Table 1:** Saponin composition of PGW.

No.	RT (min)	Compounds	Content (μg/g extract, d.b.)
1	22.37	Platycoside G1	292.56 ± 14.26
2	23.05	Platycoside E	801.72 ± 29.32
3	25.91	Deapio-platycodin D3	N.D.
4	26.82	Platycodin D3	270.99 ± 13.04
5	32.46	Deapio-platycodin D	84.45 ± 6.44
6	33.17	Platyconic acid A	194.65 ± 9.57
7	33.29	Platycodin D2	326.03 ± 23.28
8	33.63	Platycodin D	381.77 ± 13.68
9	34.81	Polygalacin D	337.76 ± 17.46
10	37.98	Platycoside A	N.D.
11	47.75	Platycodin A	N.D.
12	50.97	Platycodigenin	N.D.

Data are expressed as mean ± standard deviation of triplicate samples. N.D., not detected.

### 3.2 Cytotoxicity of PGW extract

**Figure 2:**
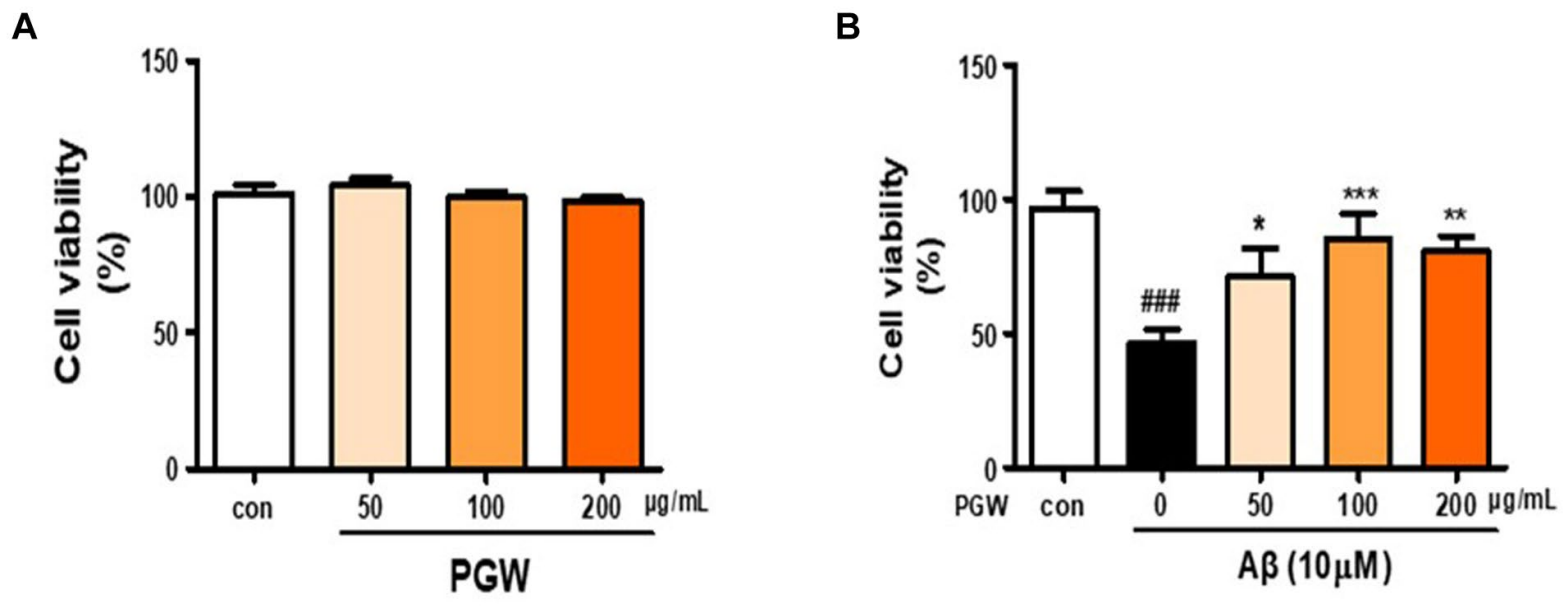
Inhibitory effect of PGW on Aβ25-35-induced oxidative stress in BV2 cells. **(A)** BV2 cells were treated with PGW (50, 100, and 200 μg/mL) for 24 h. Control groups were treated with the same volume of conditioned medium (0.2% DMSO). **(B)** BV2 cells were treated with PGW (50, 100, and 200 μg/mL) and incubated with 10 μM Aβ for 24 h Significance was determined by one-way ANOVA. *Post-hoc* analysis was performed using Tukey’s multiple comparison test; ###*p* < 0.001 compared with the control (white bar). *p < 0.05, **p < 0.01, ***p < 0.001 compared with Aβ treatment (black bar).

To assess its impact on BV2 microglial cells, the cell culture medium was treated with PGW extract at 0, 50, 100, and 200 μg/mL concentrations, followed by a MTS assay. No cytotoxic effects were observed across all concentrations of PGW extract (Figure 2A). Therefore, 200 μg/mL PGW extract was selected for subsequent experiments. In the next phase, BV2 cells were exposed to PGW at 50, 100, and 200 μM/mL concentrations for 1 h before being subjected to 10 μM of Aβ for 24 h. MTS assays revealed a reduction in cytotoxicity to approximately 46% following incubation with 10 μM amyloid beta for 24 h (Figure 2B). However, adding PGW effectively mitigated the cytotoxic effects induced by Aβ, indicating that PGW suppresses Aβ-induced cytotoxicity.

### 3.3 Inhibition of Aβ-induced ROS generation by PGW

**Figure 3:**
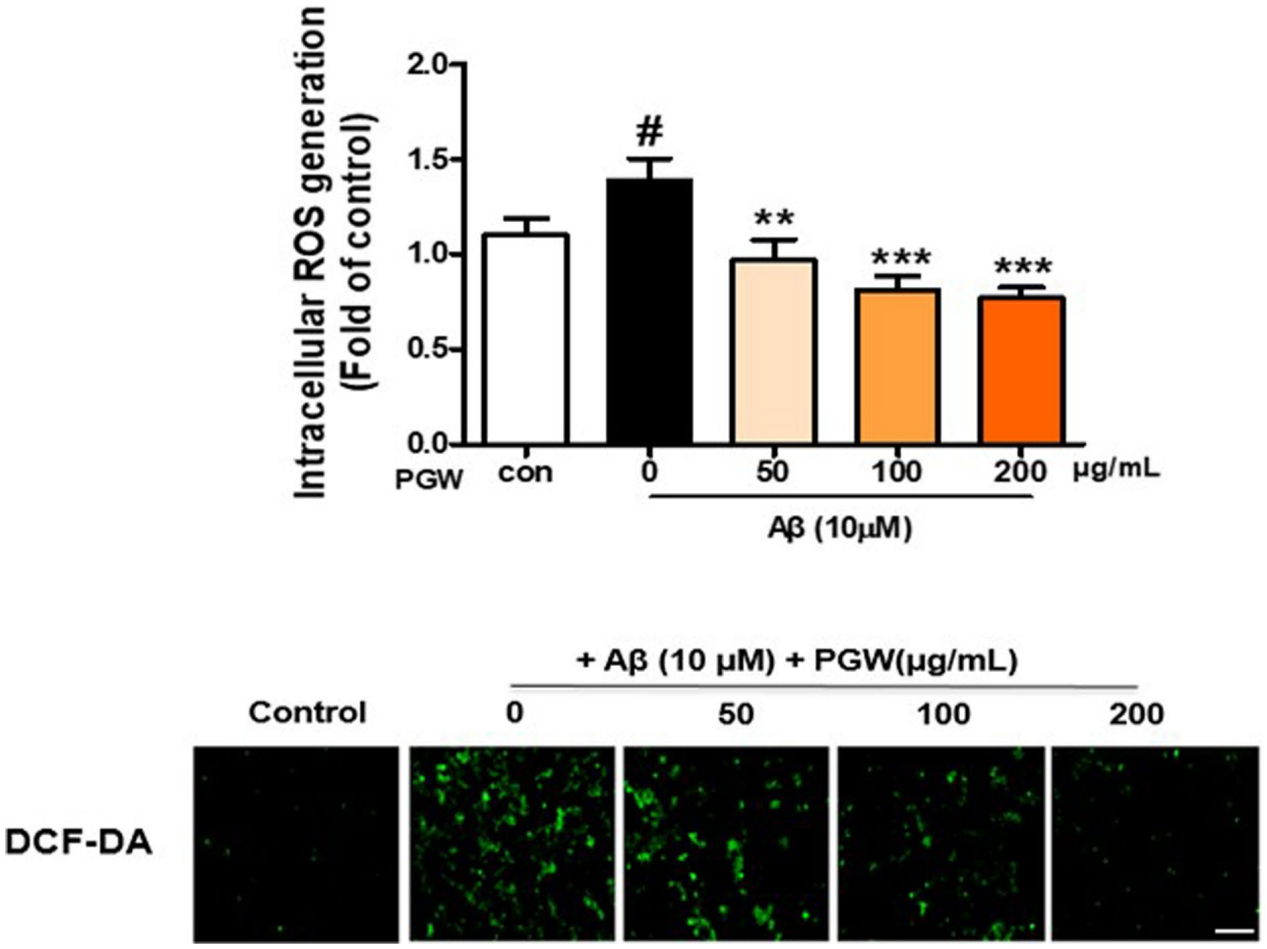
Inhibition of Aβ25-35-induced oxidative stress in BV2 cells by PGW. Cells were treated with PGW (50, 100, and 200 μg/mL) for 24 h and stimulated with Aβ25-35 (10 μM) for 30 min. Control groups were treated with the same volume of conditioned medium (0.2% DMSO). ROS generation in BV2 cells was visualized by confocal fluorescence microscopy. Absorbance was measured after DCF-DA staining of cells treated with Aβ25-35 alone, PGW (50, 100, 200 μg/mL) alone, or Aβ25-35 plus PGW. Scale bar = 50 μm. Significance was determined by one-way ANOVA. *Post-hoc* analysis was performed using Tukey’s multiple comparison test; #*p* < 0.05 compared with the control (white bar); **p < 0.01, ***p < 0.001 compared with Aβ25-35 treatment (black bar).

ROS synthesis induced by Aβ in microglia is implicated in oxidative neuronal damage and neurodegeneration, contributing to the onset of neurological diseases. Hence, we explored whether the anti-inflammatory effect of PGW was attributable to decreased ROS production. As anticipated, treatment with 10 μM Aβ markedly increased ROS synthesis (Figure 3). However, pretreatment with PGW significantly attenuated ROS levels in a dose-dependent manner, reducing them to 30.4, 42.0, and 44.2%. This suggests that PGW exerts its anti-inflammatory action by suppressing ROS production in BV2 cells.

### 3.4 Inhibition of nitric oxide production by PGW extract

**Figure 4:**
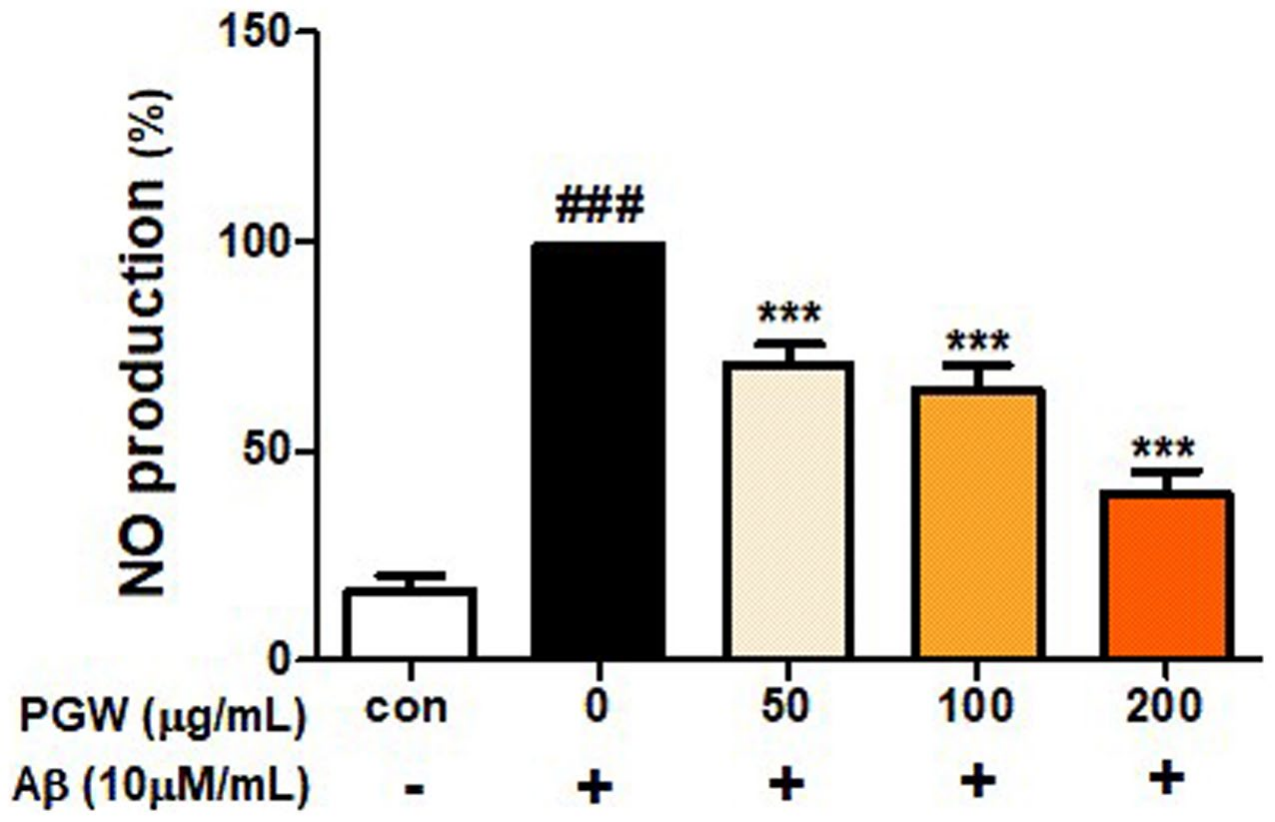
Inhibitory effects of PGW on nitric oxide production in BV2 microglia cells. The production of nitric oxide was assayed in the culture medium of cells stimulated with Aβ (10 μM) for 24 h in the presence of PGW (50, 100, and 200 μg/mL). Control groups were treated with the same volume of conditioned medium (0.2% DMSO). The result is expressed as percentages compared to the respective values obtained for the control. Data represent the means±SD with three separate experiments. Significance was determined by one-way ANOVA. *Post-hoc* analysis was performed using Tukey’s multiple comparison tests; ###*p* < 0.001 compared with the control (white bar); ***p < 0.001 compared with Aβ treatment (black bar).

Nitric oxide (NO) radical, known for its inflammatory properties, contributes to oxidative neuronal damage by reacting with oxygen to form peroxynitrite (NO3-). Thus, a considerable amount of NO, a pro-oxidant molecule that exhibits potent cytotoxicity, is generated. To validate the anti-inflammatory potential of PGW, we assessed NO production. Treatment with PGW at concentrations of 50, 100, and 200 μg/mL significantly reduced NO production by 30.4, 36.7, and 61.2%, respectively (Figure 4).

### 3.5 Inhibits production of pro-inflammatory cytokines (TNF-α, IL-6, IL-1β)

**Figure 5:**
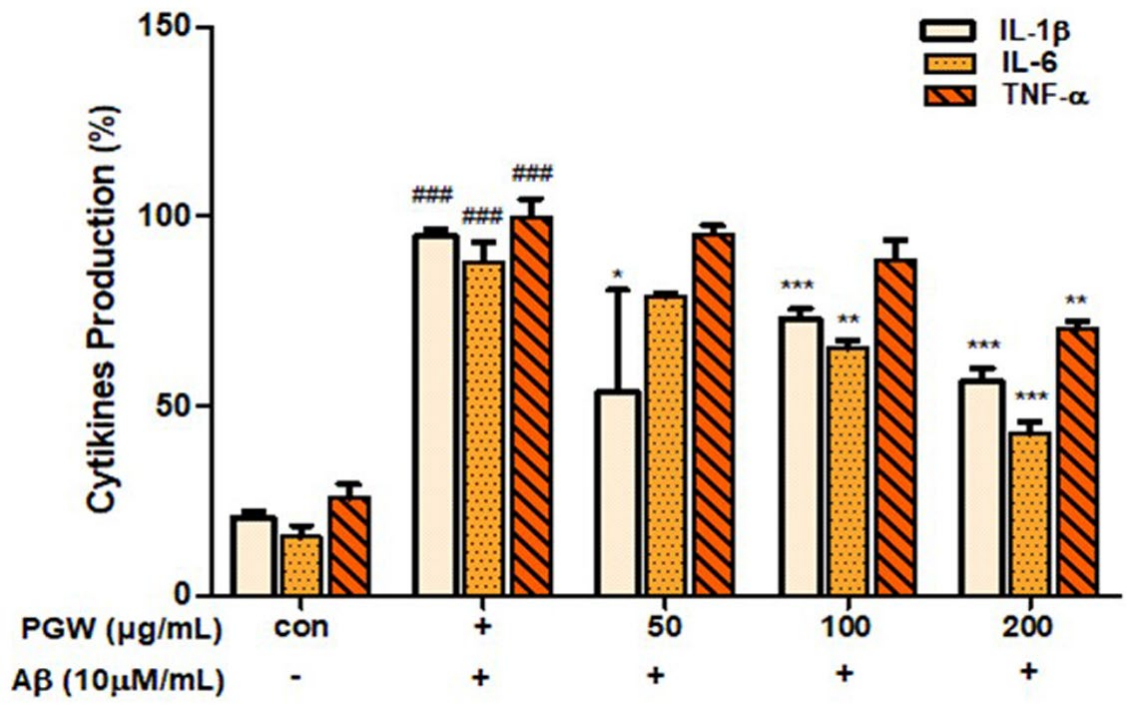
Inhibitory effects of PGW on producing pro-inflammatory cytokines in BV2 microglia cells. The production of IL-1β, IL-6, and TNF-α was assayed in the culture medium of cells stimulated with Aβ (10 μM) for 24 h in the presence of PGW (50, 100, and 200 μg/mL). Control groups were treated with the same volume of conditioned medium (0.2% DMSO). Results are expressed as a percentage of the control. Data represent the means± SD with three separate experiments. Significance was determined by one-way ANOVA. *Post-hoc* analysis was performed using Tukey’s multiple comparison tests; ###*p* < 0.001 compared with the control (white bar); *p < 0.05, **p < 0.01, ***p < 0.001 compared with Aβ treatment (black bar).

Microglial activation by Aβ leads to the secretion of various inflammatory cytokines, contributing to neuronal damage and cell death. In our experiment, we investigated the effect of PGW on the expression of cytokines IL-1β, IL-6, and TNF-α in BV2 cells induced by Aβ using an ELISA kit. Treatment with PGW at concentrations of 50, 100, and 200 μg/mL significantly suppressed the production of IL-1β by 20, 28, and 44%, respectively, and IL-6 production was significantly inhibited by 22, 35, and 58%. While TNF-α did not exhibit decrease, significant inhibitory activity was observed at 200 μg/mL (Figure 5).

### 3.6 Effect of PGW on apoptosis pathway-related factors in Aβ25-35-induced BV2 cells

**Figure 6:**
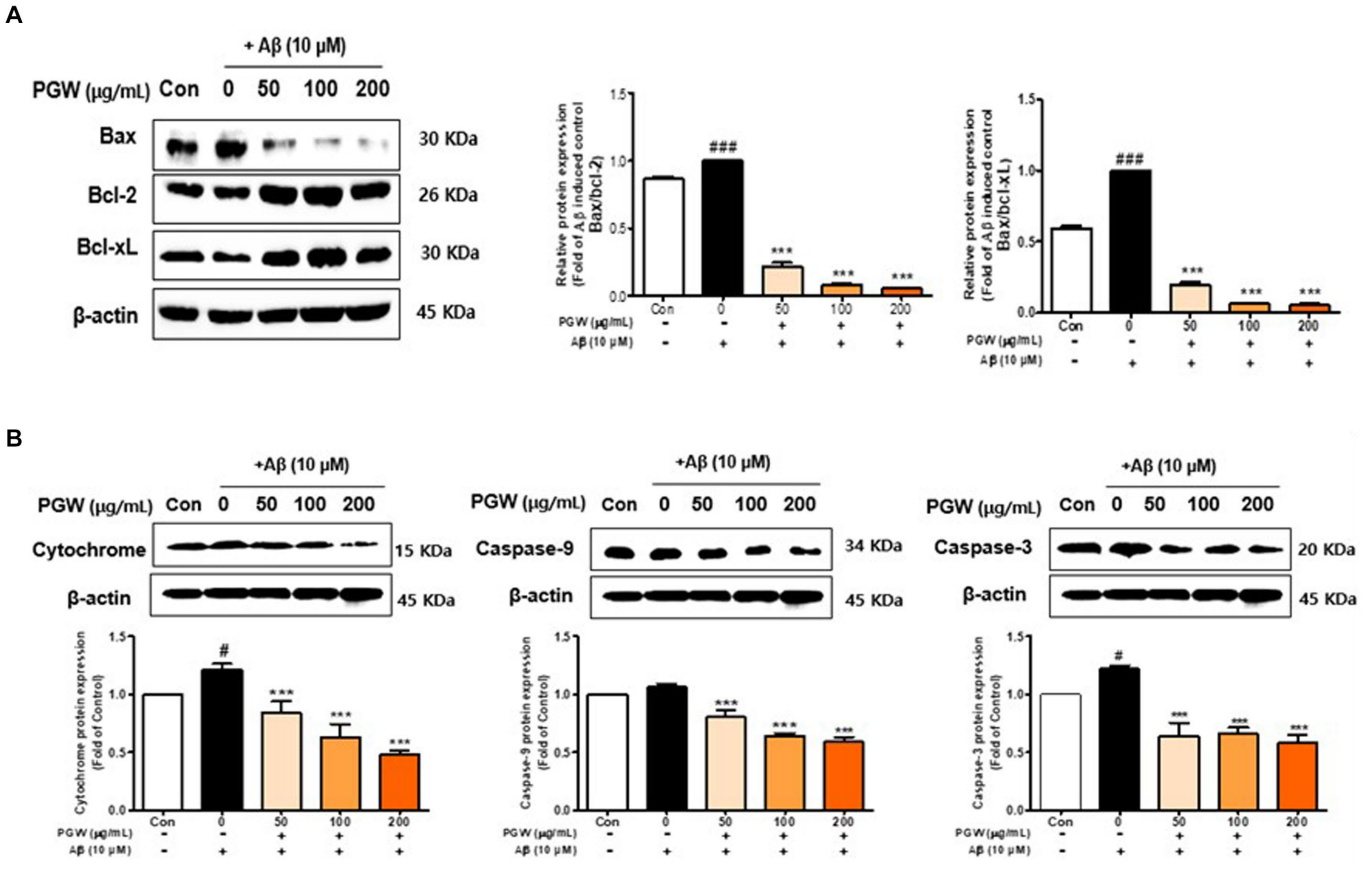
Effect of PGW on Aβ-induced Bcl-2 expression in BV2 cells: PGW protects BV2 cells from Aβ-induced oxidative apoptosis. Cells were treated with PGW (50, 100, and 200 μg/mL) for 24 h, followed by Aβ (10 μM) for 24 h. Control groups were treated with the same volume of conditioned medium (0.2% DMSO). Western blotting was performed with β-actin as the loading control. **(A)** Bax/Bcl-xL, Bax/Bcl-2, **(B)** cytochrome C, caspase-9, and caspase-3 levels in mitochondria. Values are means ± SD (*n* = 3). Significance was determined by one-way ANOVA. *Post-hoc* analysis was performed using Tukey’s multiple comparison test; #*p* < 0.05, ###*p* < 0.001 compared with the control (white bar); **p < 0.01, ***p < 0.001 compared with Aβ treatment (black bar).

We investigated whether Aβ treatment induces apoptosis in BV2 cells. As depicted in Figure 5, cells treated with Aβ exhibited a significant increase in apoptotic cells compared to control cells. Bax levels were elevated, and Bcl-2 and Bcl-xL levels were decreased in BV2 cells treated solely with Aβ. Caspase-3 activation was highest in the group administered Aβ alone (Figures 6A,B). Co-treatment of BV2 cells with Aβ and PGW significantly mitigated apoptosis compared to treatment with Aβ alone. PGW pretreatment restored Bcl-2 family protein levels similar to controls (Figure 6A). Additionally, PGW significantly inhibited caspase-3 activation in a dose-dependent manner, leading to cytochrome C release and reduced expression of caspase-9 and caspase-3 (Figure 6B). Therefore, PGW mediated inhibition of Aβ-induced apoptosis exerts neuroprotective effects by suppressing the mitochondrial apoptosis pathway and regulating Bcl-2 synthesis.

### 3.7 Effect of PGW on MAPK (JNK, ERK, p38) pathway activation in BV2 cells

**Figure 7:**
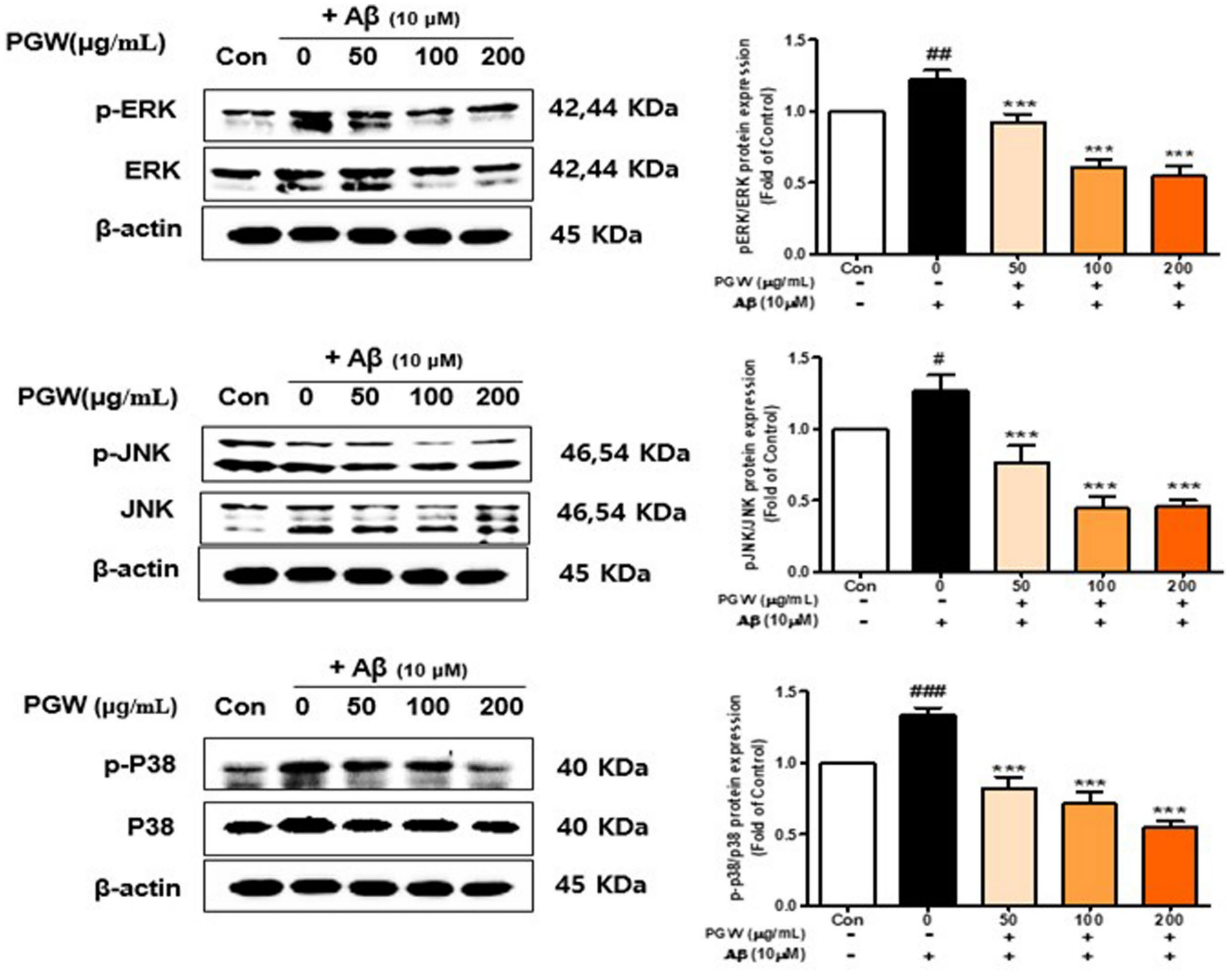
Effect of PGW on MAPK, ERK /p-ERK, JNK/p-JNK, and P38/p-P38 protein levels. Cells were treated with PGW (50, 100, and 200 μg/mL) for 24 h, followed by Aβ (10 μM) for 24 h. Control groups were treated with the same volume of conditioned medium (0.2% DMSO). Western blotting was performed using β-actin as the loading control. Values are means ± SD (*n* = 3). Significance was determined by one-way ANOVA. *Post-hoc* analysis was performed using Tukey’s multiple comparison test; #*p* < 0.05, ###*p* < 0.001 compared with the control (white bar); *p < 0.05, **p < 0.01, ***p < 0.001 compared with Aβ treatment (black bar).

To elucidate the link between PGW’s anti-inflammatory activity and the MAPK pathway, we analyzed the phosphorylation of MAPK pathway proteins. Our findings demonstrated that Aβ significantly increased the phosphorylation levels of p38, JNK, and ERK in a concentration-dependent manner. PGW treatment mitigated this increase in phosphorylation concentration independently (Figure 7). Specifically, PGW significantly decreased p38 phosphorylation at a concentration of 200 μg/mL (Figure 7). Moreover, PGW reduced JNK phosphorylation in a concentration dependent manner and ERK phosphorylation starting from the 100 μg/mL treatment group. These results suggest that PGW attenuates the inflammatory response by inhibiting the MAPK pathway in Aβ-induced BV2 microglia cells.

### 3.8 Effect of PGW on NF-κB pathway activation in BV2 cells

**Figure 8:**
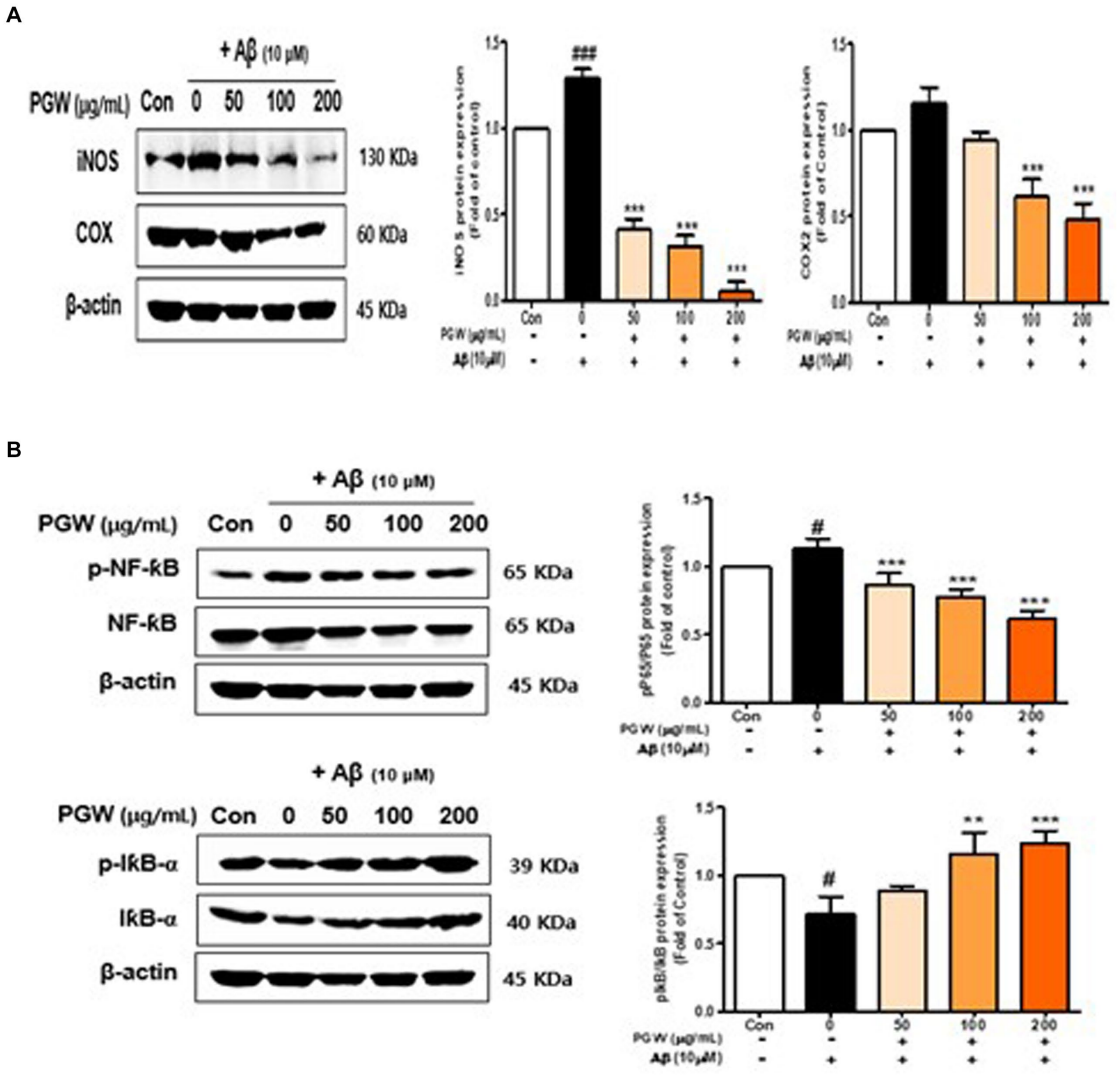
Effect of PGW on the NF-κB level in Aβ-induced cells. **(A)** iNOS and COX-2 levels. **(B)** p-NF-κB, NF-κB, I-κBα, and p-I-κBα levels. Cells were treated with PGW (50, 100, and 200 μg/mL) for 24 h, followed by Aβ (10 μM) for 24 h. Control groups were treated with the same volume of conditioned medium (0.2% DMSO). Western blotting was performed using β-actin as the loading control. Values are means ± SD (*n* = 3). Significance was determined by one-way ANOVA. *Post hoc* analysis was performed using Tukey’s multiple comparison tests; #*p* < 0.05 compared with the control (white bar); **p < 0.01, ***p < 0.001 compared with Aβ treatment (black bar).

We investigated the association between PGW’s anti-inflammatory activity and the NF-κB pathway by analyzing the expression and phosphorylation of NF-κB pathway proteins. Aβ treatment increased the phosphorylation levels of p65 and IκBα, proteins of the NF-κB pathway, whereas PGW treatment decreased their phosphorylation levels in a concentration-dependent manner (Figure 8A). Specifically, p65 phosphorylation decreased concentration independently, with a significant decrease observed at 200 μg/mL. Similarly, IκBα phosphorylation decreased concentration-dependently, reaching levels higher than those of the vehicle-treated group at 200 μg/mL (Figure 8B). These findings suggest that PGW suppresses the expression of pro-inflammatory genes by inhibiting the NF-κB pathway.

### 3.9 Dose–response curves of PGW on protein expression

This graph shows the results of analyzing the effect of PGW on the expression of various proteins by concentration. The x-axis of the graph represents the PGW concentration (μg/mL), the y-axis represents the protein expression level as a ratio compared to the control group, and each curve represents the change in expression of a specific protein.When PGW concentration exceeds 100 μg/mL, most proteins’ expression levels either plateau or increase slightly. In research and clinical applications, a PGW concentration of approximately 100 μg/mL is most effective in maximally inhibiting protein expression. Proteins such as Bax/Bcl-2 and Bcl-xL have a more robust response to PGW, significantly reducing their expression levels. This means that these proteins are more sensitive to the effects of PGW. We show that PGW may be essential in suppressing the expression of proteins that cause inflammation and apoptosis. These properties of PGW have confirmed its potential as a neuroprotective and anti-inflammatory treatment.

## 4 Discussion

PG is used as a traditional herbal medicine to treat respiratory ailments, including cough, phlegm, sore throat, and lung abscess. The bioactive components responsible for these therapeutic effects have been identified, and the potential mechanisms include inhibition of airway mucus hypersecretion, relief of inflammatory responses, and inhibition of inflammatory cytokine secretion (18, 19). AD research has recently focused on inflammation-related processes. When inflammation or nerve damage occurs, microglia are activated to produce inflammatory mediators and cytokines, thereby promoting the inflammatory response (20). Activation of microglia can induce neurotoxicity by triggering the production of proinflammatory and cytotoxic factors in neuronal cell lines treated with LPS, β-amyloid, glutamate, and arachidonate (21). Therefore, drugs that promote the neuroinflammatory response of microglia or eliminate Aβ_25–35_ may have therapeutic potential for neurodegenerative diseases. Our previous study showed that PG saponins effectively suppress Aβ-induced oxidative stress and apoptosis by activating the antioxidant pathway (Nrf2/ARE). In this study, we investigated the inhibitory effect of PGW on the production of Aβ_25–35_-induced inflammatory mediators and cytokines in BV2 microglial cells. Aβ_25–35_ is a short fragment of the amyloid precursor protein and has a neurotoxic effect similar to Aβ_1-40_ or Aβ _1–42_. Therefore, Aβ_25–35_ is suitable for studying Aβ_25-35_-induced cytotoxicity in mouse microglial cells (22). Oxidative stress is implicated in a variety of pathological processes, including cancer, diabetes, steatohepatitis, atherosclerosis, neurological degeneration, and inflammatory diseases, so we investigated the oxygen-radical scavenging activity of PG (23). There is a close relationship among oxidative stress, aging, and inflammation. Elevated levels of biomarkers for oxidative stress are associated with elevated levels of inflammatory cytokines. Calabrese et al. (24) showed that peripheral or central nerve stimulation induces inflammatory changes leading to PD symptoms and progression. Kim et al. (25) reported that an ethanol extract of PG showed significant DPPH and ABTS radical-scavenging activities and contained high levels of antioxidant phenolic compounds. Serafini suggested that the interactions of polyphenols, phenolic acids, saponins, and triterpenoids regulate NF-κB (26). Jang et al. (27) reported that several platycodin saponins from bellflower seeds inhibit the expression of proinflammatory genes by blocking NF-κB activation in LPS-induced RAW 264.7 cells. Our results show that PGW exerts an anti-inflammatory effect as a result of its antioxidant activity. In a follow-up study, we plan to investigate the anti-inflammatory activities of four selected indicators.

Early chemical studies on *P. grandiflorum* indicated that the triterpenoid saponins are the major active chemical constituents, and many previous reports suggested platycodin D and polygalacin D as the major compounds and a study by Lu et al., for individual saponin content of, platycoside E was reported to be the most prevalent compound in some provinces (28). Yoo et al., reported that platycoside E(α) was the most abundant, followed by polygalacin D2(β) and 3′-O-acetylplatyconic acid A as a result of the analysis of 18 platycosides in bellflower samples from 8 provinces in Korea (29). Our research results also showed that the content of platycoside E was the highest, followed by platycodin D, polygalacin D, platycodin D2, platycoside G1, platycodin D3, platyconic acid A, and deapio-platycodin D (Figure 1; Table 1). Choi et al. reported that bellflower saponin platicoside E effectively improved ethanol-induced cognitive dysfunction in rats (30).

Polygalacin D2 and platycodin D have been reported to reduce Neuroinflammation (31). It has been reported that platycodin D2 is an adjuvant to increase Th1 and Th2 cytokines (32). In the composition of PG saponins present in PGW, it was confirmed that PGW has neuroinflammation inhibitory activity, as in the results of previous studies.

Neuroinflammation and apoptosis are related, and the inflammatory response can cause damage to nerve cells, leading to their apoptosis. Among the genes involved in regulating apoptosis, Bcl-2 and Bcl-XL have anti-apoptotic effects that promote cell survival (33). By contrast, Bax (Bcl-2-associated X protein), Bad, Bak, Bik, and Bcl-XS have proapoptotic effects. In addition, c-Jun N-terminal kinase (JNK) regulates apoptosis (34). JNK is activated by apoptotic signals and phosphorylates several intracellular factors, thereby regulating apoptosis. Neuronal injury caused by cytokines and inflammatory or heat shock-inducing factors triggers the JNK and p38 signaling pathways and induces ERK signaling, leading to oxidative stress-mediated death of neurons (35). In this study, JNK was phosphorylated, the ratio of Bax/Bcl-2 was increased, and caspase-3 was activated in BV2 microglia treated with β-amyloid (10 μM) for 24 h (Figure 6).

In our previous study, saponin, an important component of PGE, not only alleviated oxidative stress and prevented neuronal death in Aβ-treated hippocampal HT22 cells, but also alleviated AD-related pathologies such as Aβ deposition, oxidative damage, and neuroinflammation. Additionally, bellflower root extract (PGE) inhibited Aβ accumulation in the brain of 5XFAD mice. In this study, we aimed to elucidate the mechanism by which PGW alleviates neuroinflammation in Aβ-treated BV2 microglia. Subsequent studies demonstrate that PGE administration significantly inhibits the deposition of Aβ in the brains of 5XFAD mice.

MAPKs (e.g., JNK, ERK, and p38) are important regulators of the production of proinflammatory cytokines such as iNOS and COX2, including NF-κB. Nam et al. (36) reported that the upregulation of proinflammatory cytokines induced by Aβ_25-35_ was suppressed in HT22 neurons, and Ji et al. (10) showed that a bellflower saponin fraction significantly inhibited the Aβ_25-35_-induced phosphorylation of ERK1/2 MAPK, in turn inhibiting inflammatory mediator production in neurons. In this study, p-JNK and p-P38 levels were increased, and that of p-ENK was decreased, by Aβ_25-35_. By contrast, PGW ameliorated Aβ_25-35_-induced neuroinflammation by inhibiting the p38 signaling pathway in BV2 microglial cells (Figure 7). Although the roles of MAPKs in cytokine synthesis vary depending on the cytokine, and synthesis can occur at different steps in the signaling cascade in the same cell, our data suggest that PGW has potential as an anti-inflammatory and neuroprotective agent.

We confirmed that Aβ_25-35_-induced PGW significantly increased the production of IL-1β, IL-6, and TNF-α in Aβ_25-35_-treated BV2 microglial cells. On the other hand, TNF-α did not show a significant decrease, but significant inhibitory activity was confirmed at 200 μg/mL (Figure 5). It is associated with the regulation of transcriptional activity. Jang et al. (27) reported that a water extract of *P. grandiflorum* root upregulated iNOS and TNF-α and transcriptionally activated NF-κB in LPS-stimulated RAW 264.7 cells. Ahn et al. showed that *P. grandiflorum* saponins exert anti-inflammatory effects by inhibiting the LPS-induced expression of iNOS and COX-2 by blocking NF-κB activation in RAW 264.7 macrophages (37). In this study, PGW significantly suppressed the activation of NF-κB by Aβ and increased the expression of I-κBα (Figure 8B). PGW also inhibited NF-κB signaling by regulating the activation of NF-κB and IκBα. Additionally, the levels of iNOS and COX-2 were significantly decreased by PGW compared to Aβ alone (Figure 8A). Therefore, PGW attenuated Aβ-induced neuroinflammation by downregulating NF-κB signaling.

**Figure 9:**
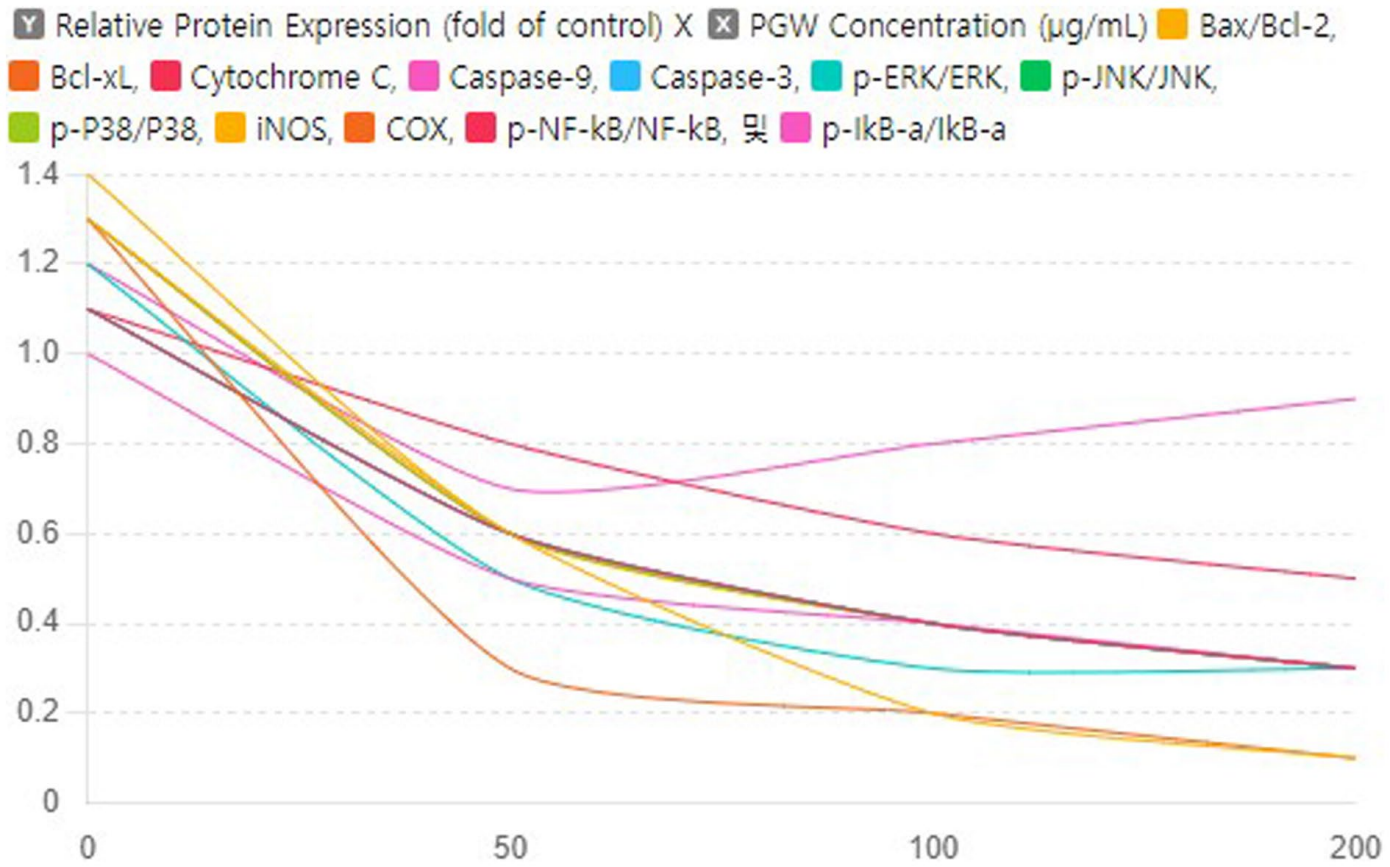
Impact of PGW on Protein Expression in Response to Aβ: Dose–Response Curves. Nonlinear regression analysis was performed to derive curves showing the relationship with PGW concentration for each protein expression level. Significance was determined by one-way ANOVA. *Post-hoc* analysis was performed using Tukey’s multiple comparison tests; PGW treatment at 50, 100, and 200 μg/mL generally leads to very significant changes (*p* < 0.001) compared to the control.

Based on the results of a study analyzing the effect of PGW on protein expression by concentration, the PG dose was investigated, and it was confirmed that a concentration of 100 μg/mL had the optimal effect (Figure 9). At this concentration, the expression of most proteins was maximally suppressed, and in particular, the expression of proteins that cause inflammation and apoptosis was significantly reduced. Therefore, in research and clinical applications, a PGW concentration of 100 μg/mL is most effective in maximally suppressing protein expression, providing important baseline data to increase the potential utilization of PGW as a neuroprotective and anti-inflammatory therapeutic agent.

In summary, PGW demonstrated anti-inflammatory activity by inhibiting the specific MAPK signaling pathways, including JNK and ERK, and suppressing the activation of NF-κB. This inhibition resulted in the downregulation of iNOS and COX-2 expression, leading to a reduction in the production of inflammatory mediators and pro-inflammatory cytokines. Based on these findings, PGW has been identified as a valuable material for effectively preventing and treating neurodegenerative inflammatory disorders through its anti-inflammatory regulatory efficacy.

## 5 Conclusion

**Figure 10:**
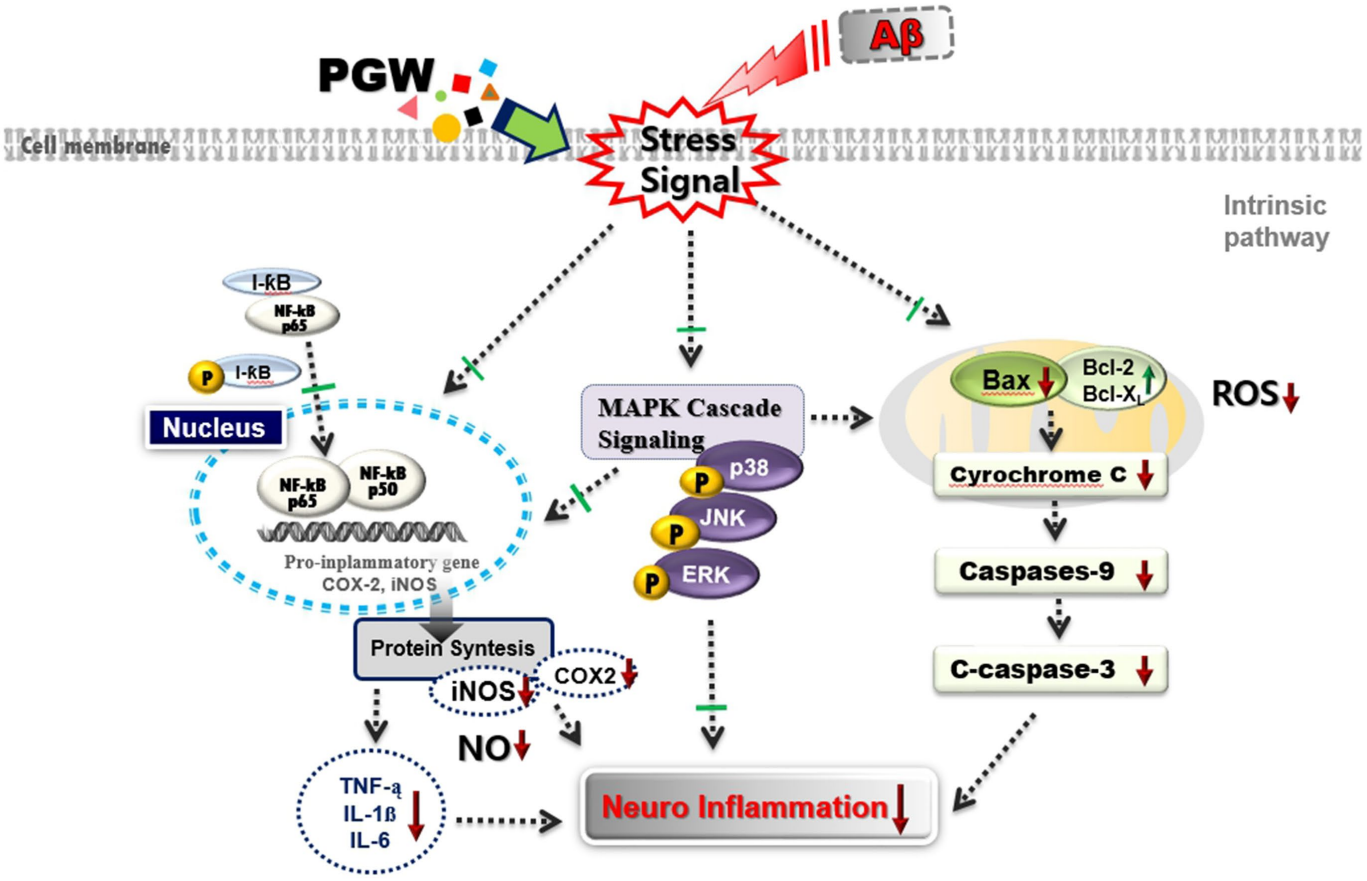
Schematic diagram of the mechanism of Anti-neuroflammatory effects of PGW in Aβ-stimulated BV2 microglia. PGW regulates Aβ toxicity-induced inflammatory response by inhibiting MAPK and NF-κB activation in BV2 microglia. ↑, increase or up-regulation; ↓, decrease or down-regulate; ⊥, block or inhibit.

As shown in the schematic diagram of Figure 10, PGW demonstrated anti-inflammatory activity by inhibiting the specific MAPK signaling pathways, including JNK and ERK, and suppressing the activation of NF-κB. This inhibition resulted in the downregulation of iNOS and COX-2 expression, leading to a reduction in the production of inflammatory mediators and pro-inflammatory cytokines. Based on these findings, PGW has been identified as a valuable material for effectively preventing and treating neurodegenerative inflammatory disorders through its anti-inflammatory regulatory efficacy.

## Data availability statement

The datasets presented in this study can be found in online repositories. The names of the repository/repositories and accession number(s) can be found in the article/Supplementary material.

## Ethics statement

Ethical approval was not required for the studies on animals in accordance with the local legislation and institutional requirements because only commercially available established cell lines were used.

## Author contributions

Y-JJ: Writing – original draft, Writing – review & editing. MK: Formal analysis, Software, Writing – review & editing. G-SK: Conceptualization, Data curation, Writing – review & editing. HK: Conceptualization, Project administration, Writing – review & editing. GJ: Methodology, Writing – review & editing.
