# Supplementary figures and images for: Platycodon grandiflorum exhibits anti-neuroinflammatory potential against beta-amyloid-induced toxicity in microglia cells
Source: Front Nutr. 2024 Aug 7;11:1427121. doi: 10.3389/fnut.2024.1427121 (PMC11335668; doi:10.3389/fnut.2024.1427121)

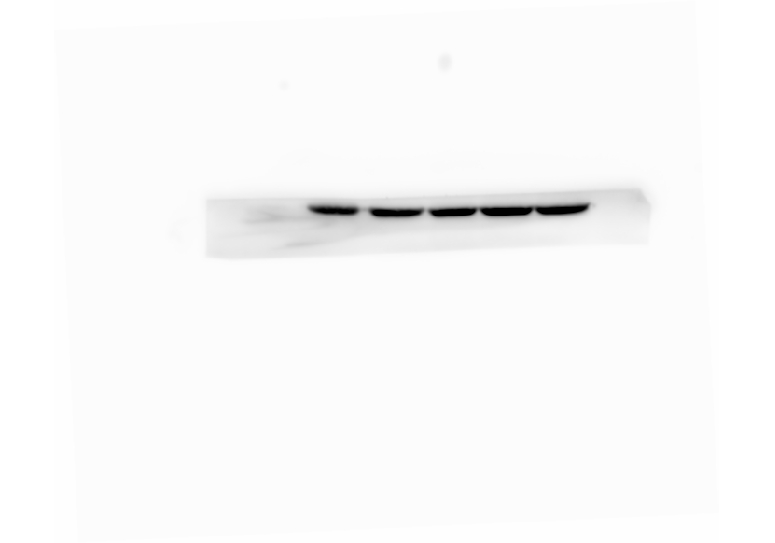

Supplement: Supplementary file 1 [file Data_Sheet_1.ZIP › Fig 6.Apoptosis -western/Actin.jpg]

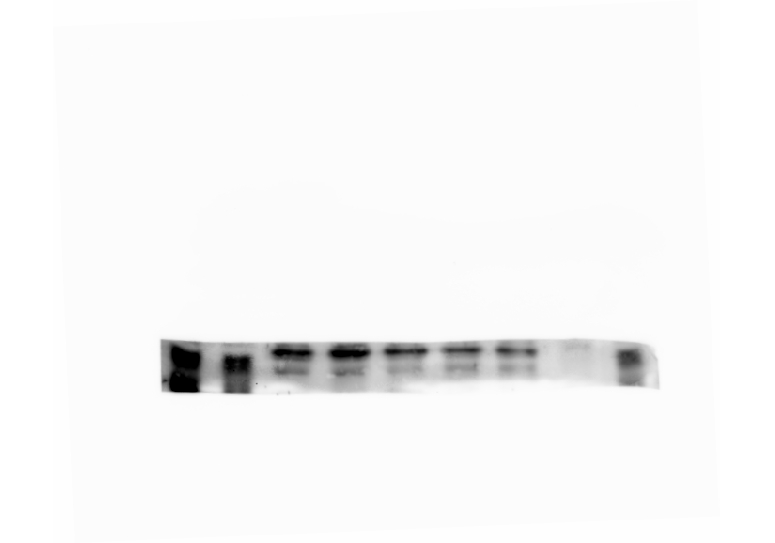

Supplement: Supplementary file 1 [file Data_Sheet_1.ZIP › Fig 6.Apoptosis -western/BAX.jpg]

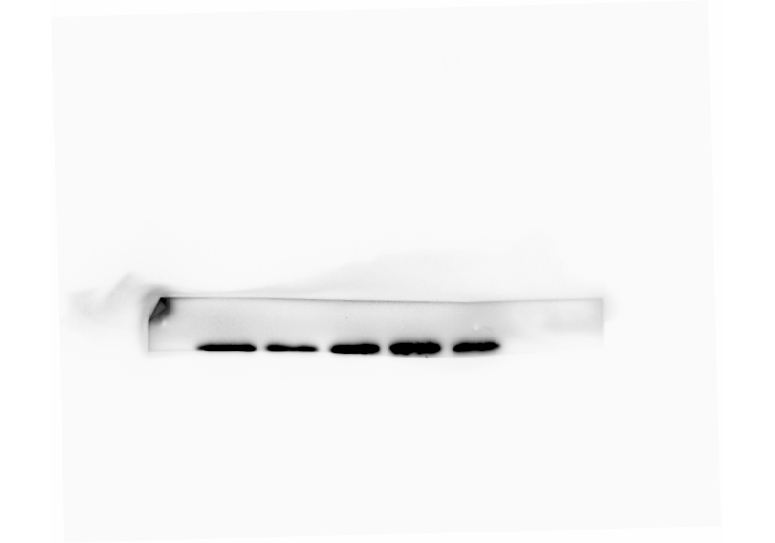

Supplement: Supplementary file 1 [file Data_Sheet_1.ZIP › Fig 6.Apoptosis -western/Bcl-xL.jpg]

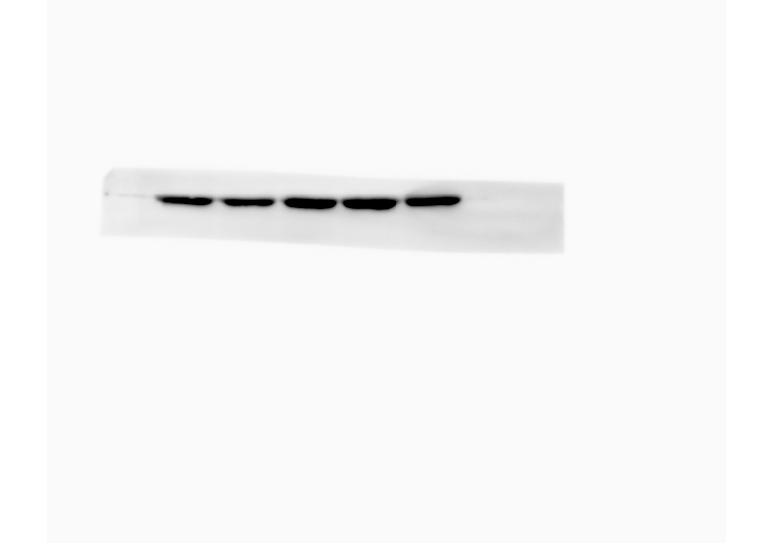

Supplement: Supplementary file 1 [file Data_Sheet_1.ZIP › Fig 6.Apoptosis -western/Bcl2.jpg]

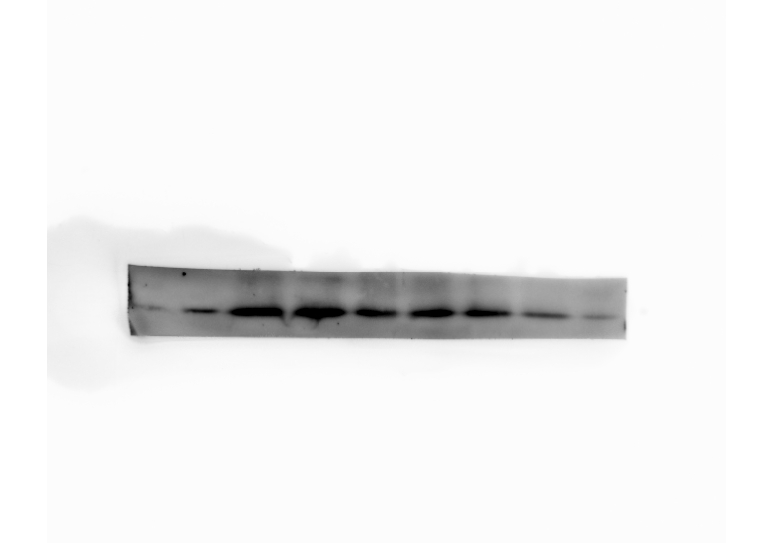

Supplement: Supplementary file 1 [file Data_Sheet_1.ZIP › Fig 6.Apoptosis -western/Caspase3.jpg]

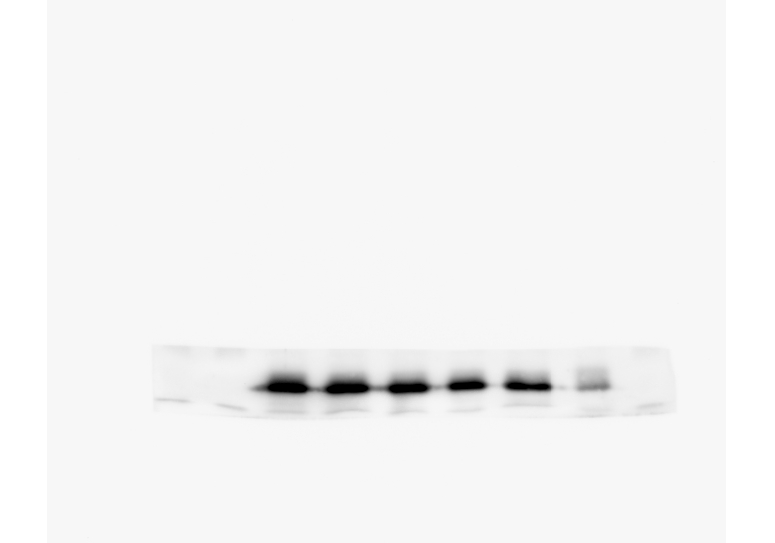

Supplement: Supplementary file 1 [file Data_Sheet_1.ZIP › Fig 6.Apoptosis -western/Caspase9.jpg]

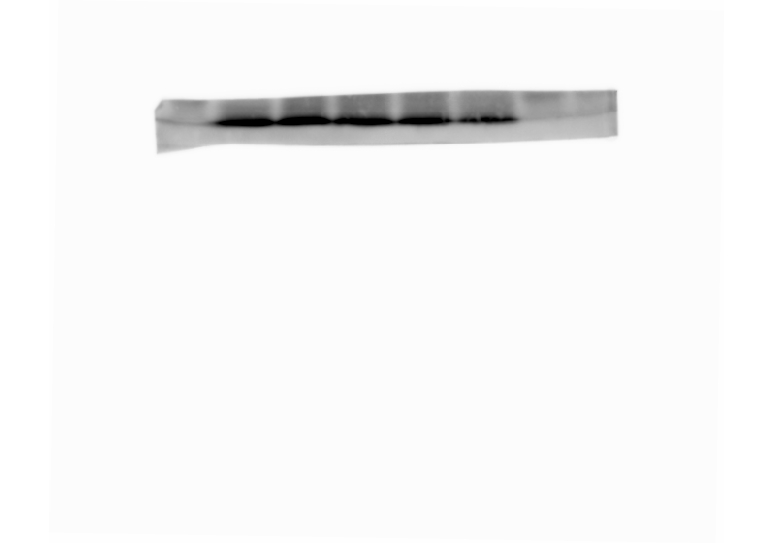

Supplement: Supplementary file 1 [file Data_Sheet_1.ZIP › Fig 6.Apoptosis -western/CytoC.jpg]

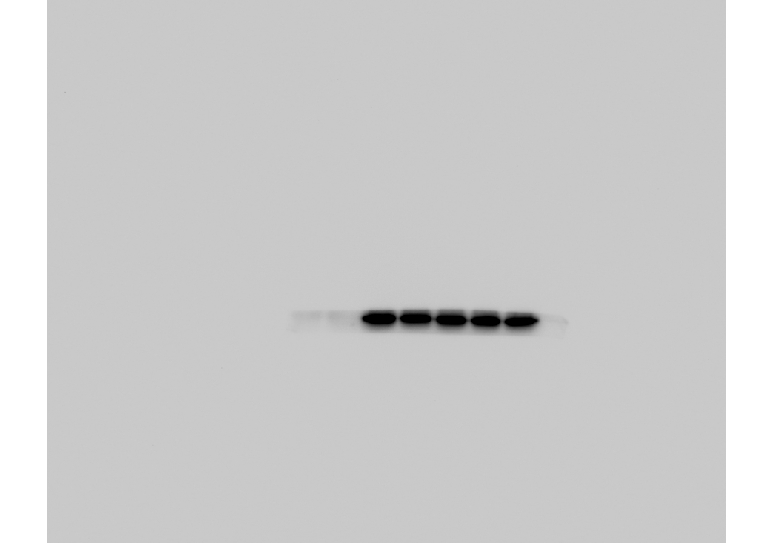

Supplement: Supplementary file 1 [file Data_Sheet_1.ZIP › Fig 7.MAPK-western/ACTIN(Chemiluminescence).jpg]

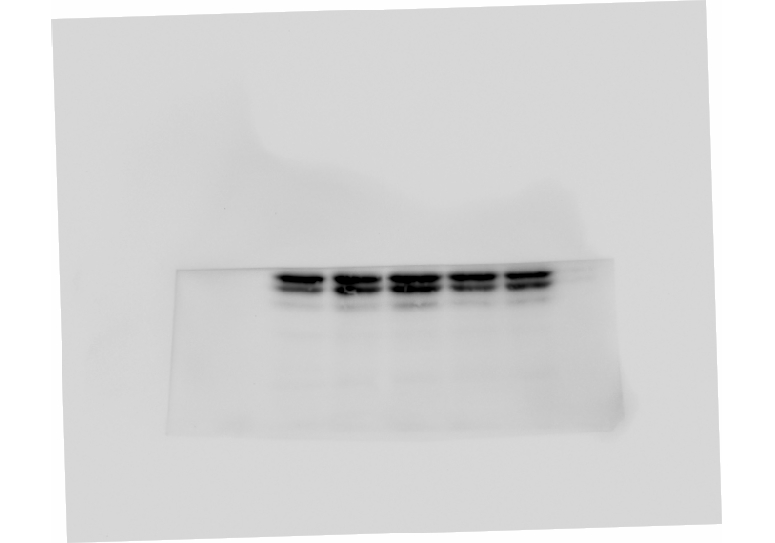

Supplement: Supplementary file 1 [file Data_Sheet_1.ZIP › Fig 7.MAPK-western/ERK(Chemiluminescence).jpg]

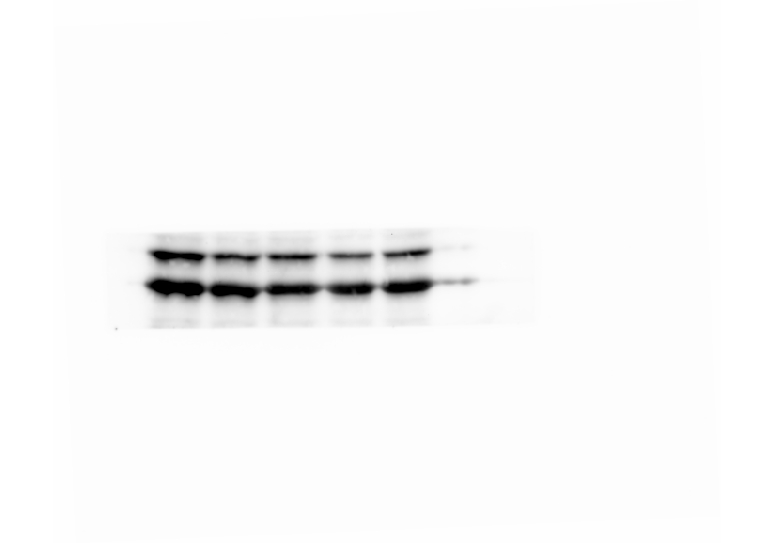

Supplement: Supplementary file 1 [file Data_Sheet_1.ZIP › Fig 7.MAPK-western/JNK.jpg]

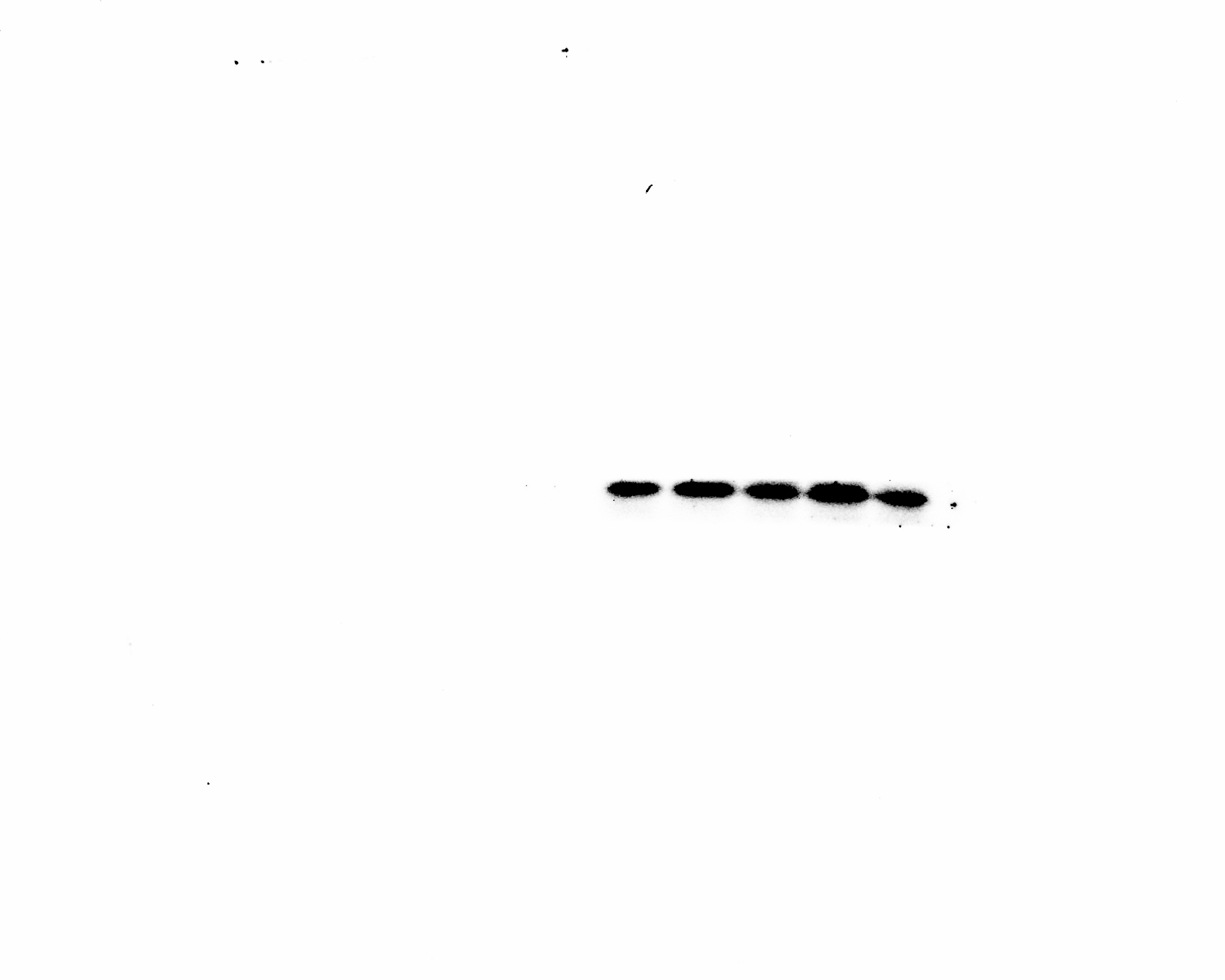

Supplement: Supplementary file 1 [file Data_Sheet_1.ZIP › Fig 7.MAPK-western/JYJ 2021-05-03 P-p38-w(Chemiluminescence).jpg]

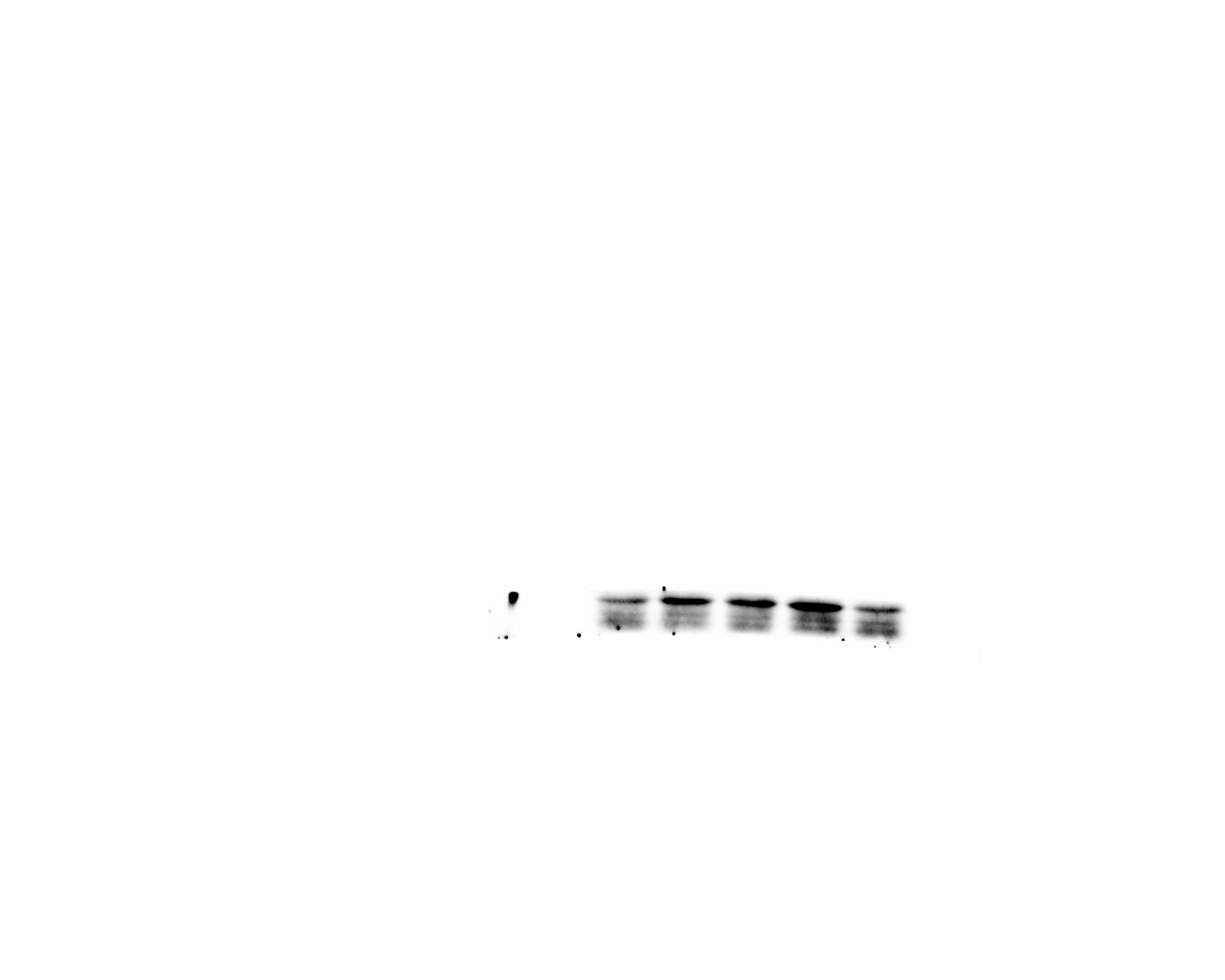

Supplement: Supplementary file 1 [file Data_Sheet_1.ZIP › Fig 7.MAPK-western/JYJ 2021-06-16 HO1-ps-2(Chemiluminescence).jpg]

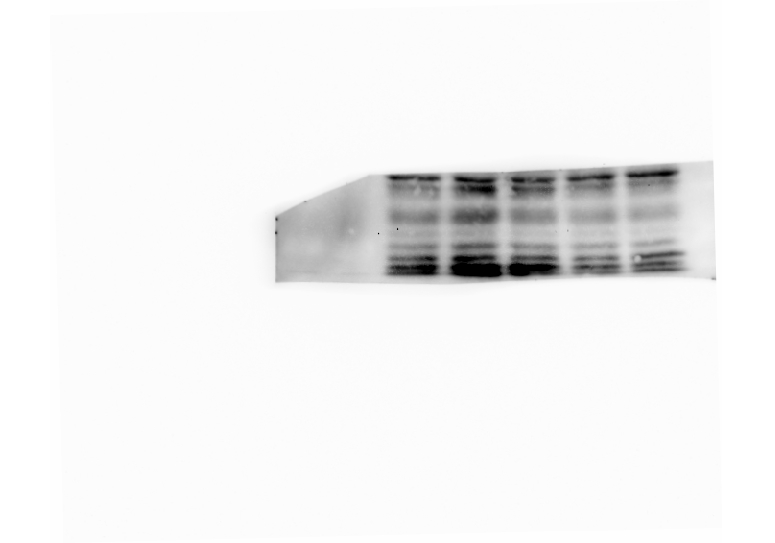

Supplement: Supplementary file 1 [file Data_Sheet_1.ZIP › Fig 7.MAPK-western/P-ERK(Chemiluminescence).jpg]

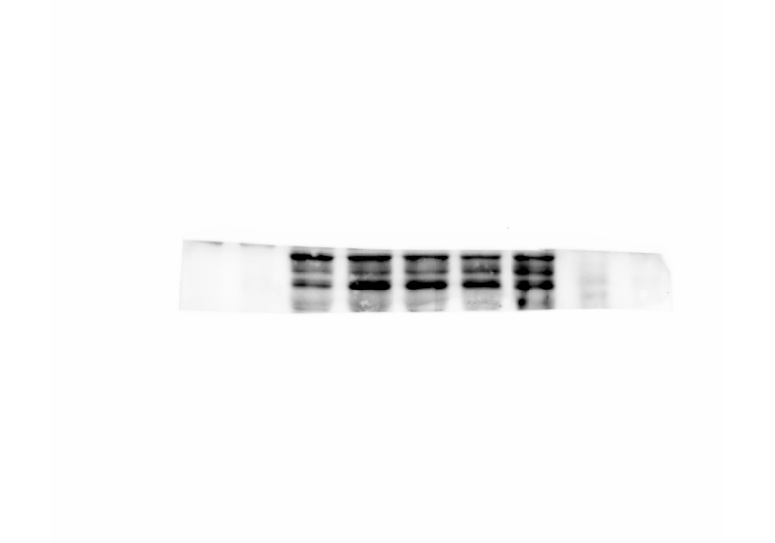

Supplement: Supplementary file 1 [file Data_Sheet_1.ZIP › Fig 7.MAPK-western/P-JNK.jpg]

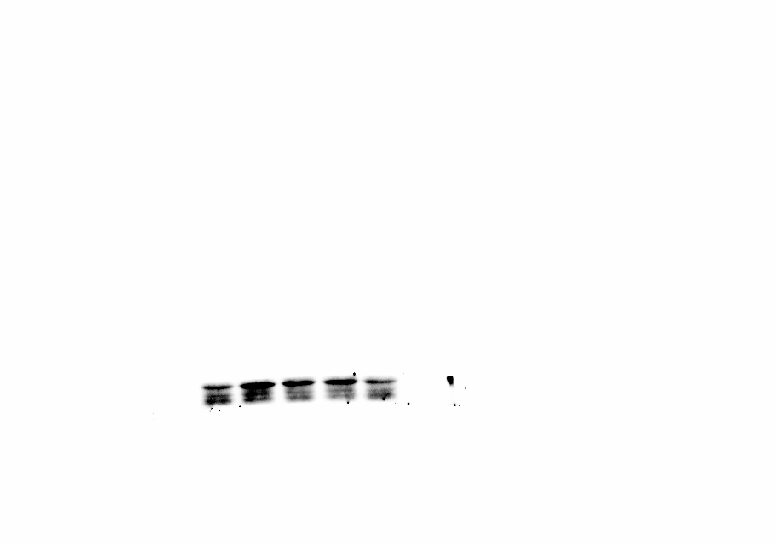

Supplement: Supplementary file 1 [file Data_Sheet_1.ZIP › Fig 7.MAPK-western/p-p38.jpg]

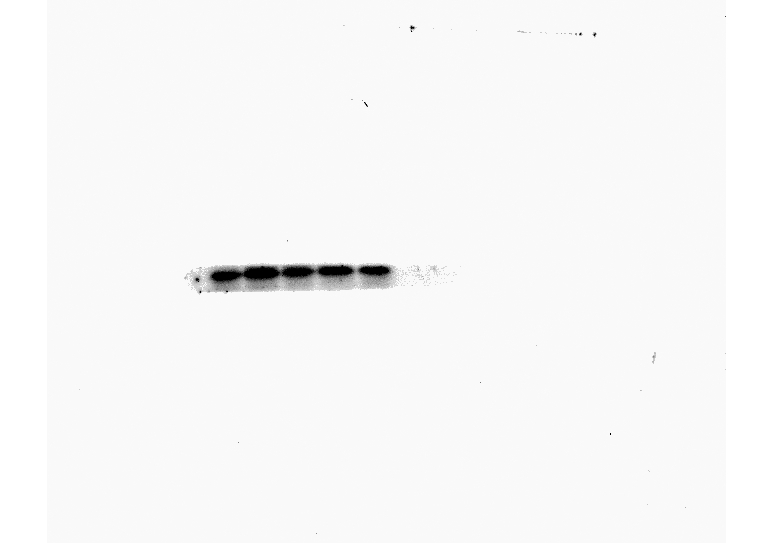

Supplement: Supplementary file 1 [file Data_Sheet_1.ZIP › Fig 7.MAPK-western/p38.jpg]

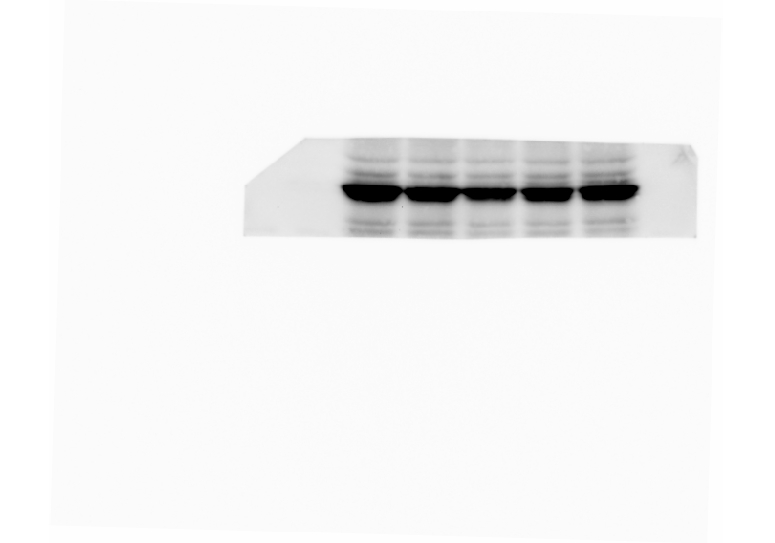

Supplement: Supplementary file 1 [file Data_Sheet_1.ZIP › Fig 8.NFKB -western/Actin-1(Chemiluminescence).jpg]

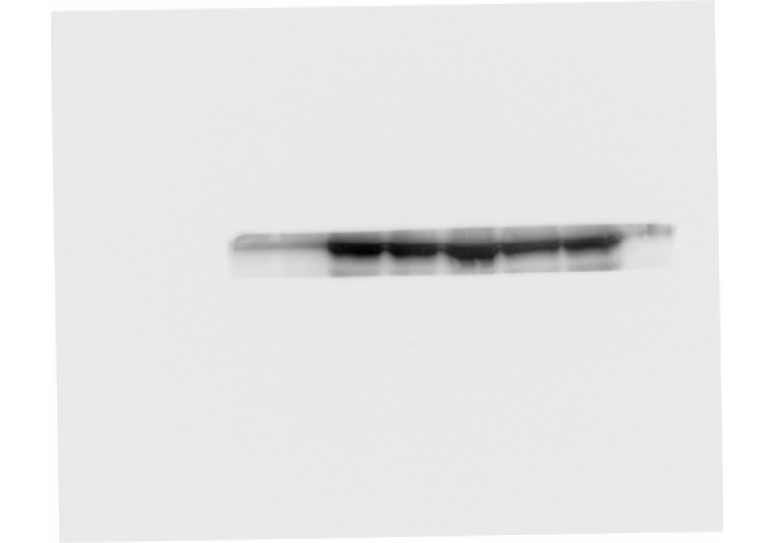

Supplement: Supplementary file 1 [file Data_Sheet_1.ZIP › Fig 8.NFKB -western/Cox-1(Chemiluminescence).jpg]

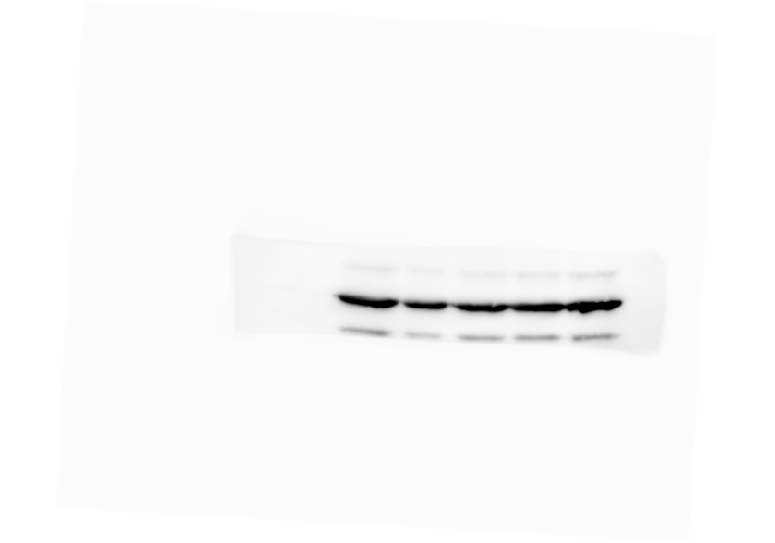

Supplement: Supplementary file 1 [file Data_Sheet_1.ZIP › Fig 8.NFKB -western/IKB(Chemiluminescence)-1.jpg]

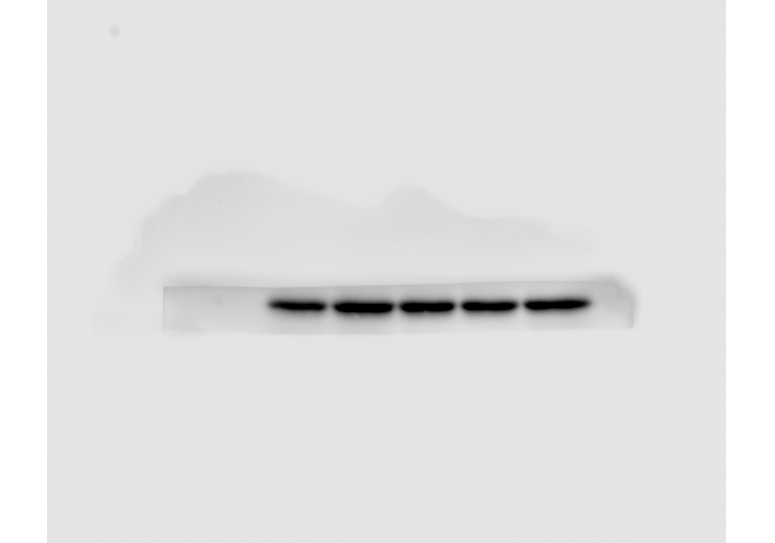

Supplement: Supplementary file 1 [file Data_Sheet_1.ZIP › Fig 8.NFKB -western/P-P65-1(Chemiluminescence).jpg]

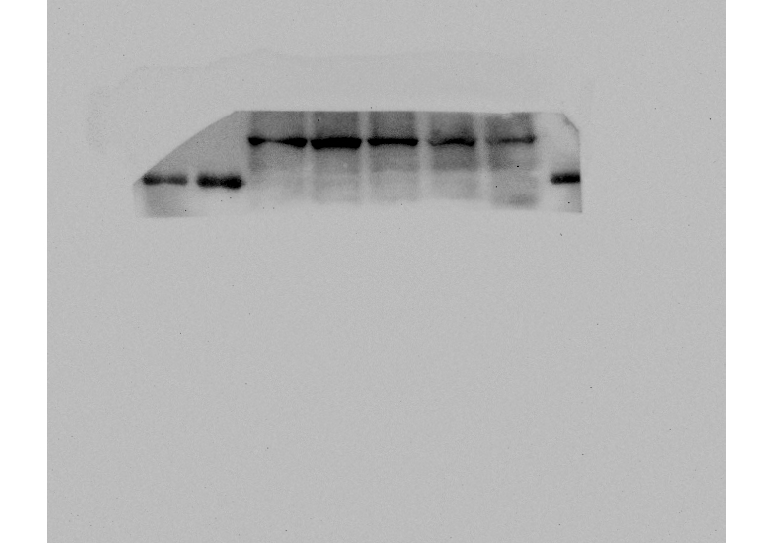

Supplement: Supplementary file 1 [file Data_Sheet_1.ZIP › Fig 8.NFKB -western/iNOS-1(Chemiluminescence).jpg]

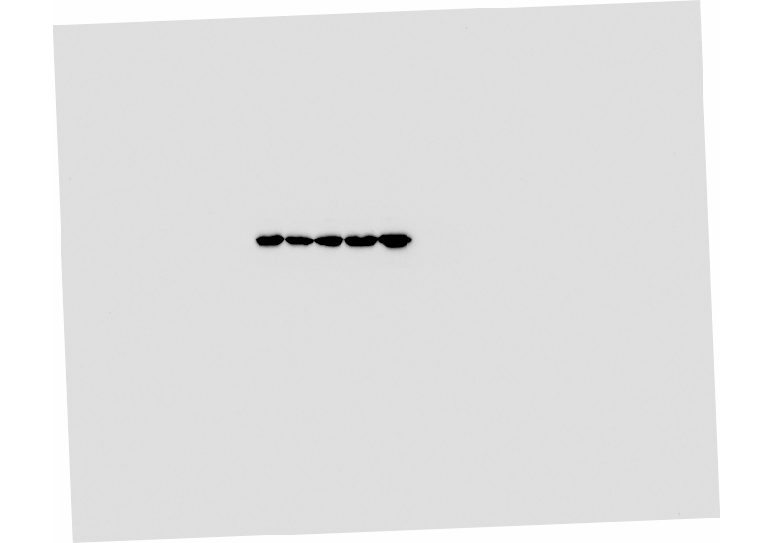

Supplement: Supplementary file 1 [file Data_Sheet_1.ZIP › Fig 8.NFKB -western/p-iKB-1.jpg]

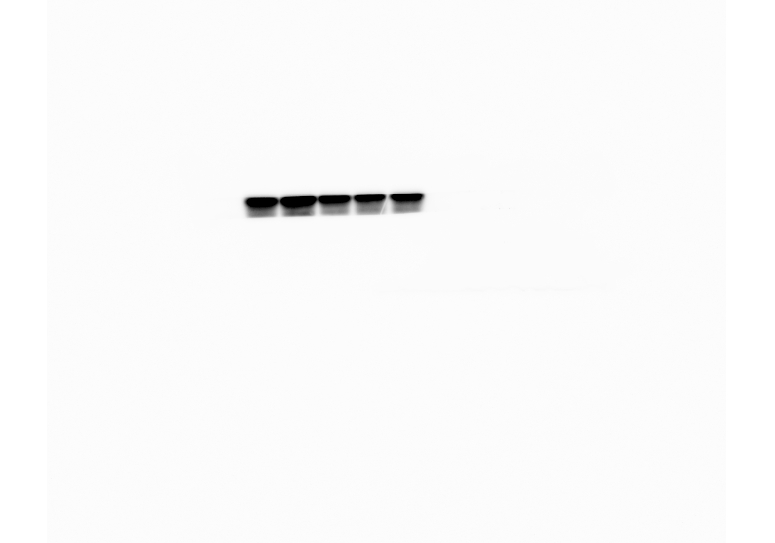

Supplement: Supplementary file 1 [file Data_Sheet_1.ZIP › Fig 8.NFKB -western/p65.jpg]
